# Exosome membrane-biomimetic nanomedicine targets the pre-metastatic niche *via* NF-κB inhibition to suppress breast cancer lung metastasis

**DOI:** 10.1016/j.mtbio.2026.102856

**Published:** 2026-01-31

**Authors:** Rui Tang, Chengyu Mao, Caofang Hu, Wei Liu, Ju Bai, Yali Wang, Lijun Yang, Hongzhao Qi

**Affiliations:** a Institute for Translational Medicine, The Affiliated Hospital of Qingdao University, College of Medicine, Qingdao University, Qingdao, 266021, China; b Qingdao Institute of Bioenergy and Bioprocess Technology, Chinese Academy of Sciences, Qingdao, 266101, China; c Department of Chemistry, College of Pharmacy, North China University of Science and Technology, Tangshan, 063210, China; d Department of Emergency Medicine, The Affiliated Hospital of Qingdao University, Qingdao, 266003, China

**Keywords:** Tumor metastasis, Pre-metastatic niche, Inflammation, PDTC, Exosome membrane

## Abstract

Breast cancer lung metastasis remains a major cause of mortality, largely driven by the formation of a pre-metastatic niche (PMN) through inflammatory signaling. Here, we report a biomimetic nanomedicine, EXO@m(PDTC), designed to target the pulmonary PMN and inhibit metastasis *via* suppression of nuclear factor-κB (NF-κB) signaling. The nanoconstruct consists of pyrrolidine dithiocarbamate (PDTC), an NF-κB inhibitor, encapsulated within micelles coated with exosome membranes derived from breast cancer cells. This design leverages the innate lung-homing ability of tumor exosomes, enabling precise accumulation in incipient PMNs. We demonstrate that EXO@m(PDTC) effectively inhibits NF-κB activation in multiple pulmonary stromal cell types, downregulates pro-inflammatory cytokines, and attenuates PMN formation. In both tail vein and orthotopic breast cancer models, EXO@m(PDTC) significantly reduces lung metastasis with minimal systemic toxicity. Transcriptomic analysis further reveals downregulation of NF-κB-associated pathways, including cytokine-cytokine receptor interaction and chemokine signaling. Our study highlights a promising strategy for intercepting metastasis through early PMN disruption and offers a targeted nanotherapeutic platform with high clinical potential.

## Graphical abstract

**Figure:**
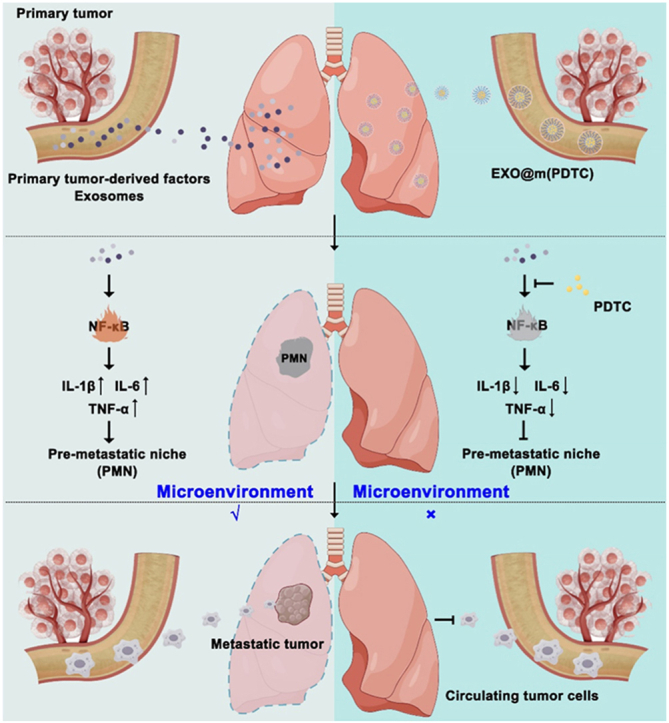

## 1 Introduction

Breast cancer is the most prevalent malignant tumor worldwide, accounting for approximately one-third of all malignancies in women, with a mortality rate of roughly 15% among diagnosed cases [1,2]. Metastasis is the primary cause of mortality in breast cancer patients, accounting for 90% of deaths, with the lungs being the most common site of dissemination (60–70% of cases) [3]. Consequently, developing strategies to inhibit lung metastasis is critical for improving breast cancer patient survival.

For an extended period, research on inhibiting tumor metastasis has primarily focused on interventions targeting the primary tumor. For instance, suppressing the epithelial-mesenchymal transition (EMT) or impairing the migratory and invasive capabilities of tumor cells at the primary site can hinder metastatic dissemination [4]. In this context, numerous nanomedicines have been developed. For example, Liu et al. designed a novel multiple-enzyme co-expressed Co-PN_3_ SA/CHO nanoagent, which disrupts the lipid raft structure and inhibits lamellipodia formation through cholesterol depletion and oxidative stress upregulation [5]. This effectively suppresses tumor cell motility, migration, and subsequent metastasis. However, due to the high heterogeneity and mutability of tumor cells, metastasis remains a persistent therapeutic challenge [6]. Consequently, solely targeting the behavior and characteristics of tumor cells proves insufficient for effective anti-metastatic therapy.

Over the past decade, research has revealed that metastasis is preceded by the construction of a pre-metastatic niche (PMN) in distant organs [7]. The primary tumor educates bone-marrow-derived cells (BMDCs) and resident stromal cells through secreted factors and exosomes, orchestrating a cascade of events, including immunosuppression, inflammation, angiogenesis, lymphangiogenesis, extracellular matrix (ECM) remodeling, and metabolic reprogramming, that collectively create a supportive “soil” for circulating tumor cells (CTCs) [8]. Targeting the molecular and cellular drivers of PMN formation is therefore a promising therapeutic strategy. For example, Zhou et al. developed an enzyme-activated peptide, FR17, which suppresses postoperative lung metastasis of melanoma by inhibiting fibroblast activation and MDSC recruitment in the PMN [9]. Despite these advances, effective inhibition of PMN requires deeper mechanistic insight and more sophisticated intervention strategies.

Inflammation serves as both the initiator and amplifier of PMN formation [10]. It induces immunosuppression by promoting the recruitment of MDSCs and regulatory T cells (Tregs) *via* cytokines such as tumor necrosis factor-α (TNF-α) and S100A8/A9 [11]. Simultaneously, inflammation synergizes with vascular endothelial growth factor (VEGF) to stimulate angiogenesis and lymphangiogenesis, facilitating tumor cell dissemination [12]. Inflammatory mediators also upregulate ECM-remodeling enzymes like lysyl oxidase (LOX) and matrix metalloproteinases (MMPs), creating “docking sites” for CTCs [13]. Furthermore, inflammation establishes chemokine gradients (e.g., CXCL1) that guide organotropism, and it triggers metabolic reprogramming to support metastatic growth [14]. Given its central role, targeting the inflammatory response represents a powerful strategy to disrupt PMN formation broadly. For instance, Jiang et al. demonstrated that hybrid micelles co-loaded with metformin and docosahexaenoic acid can modulate PMN formation and suppress metastasis by alleviating local inflammation [15].

However, effectively targeting inflammation within the PMN presents two major challenges: first, comprehensively inhibiting inflammatory signaling across diverse stromal cell types; and second, intervening at the earliest possible stage before the niche is fully established. Overcoming these hurdles requires identifying key therapeutic targets and developing delivery systems capable of precise, early accumulation at PMN sites.

**Scheme 1:**
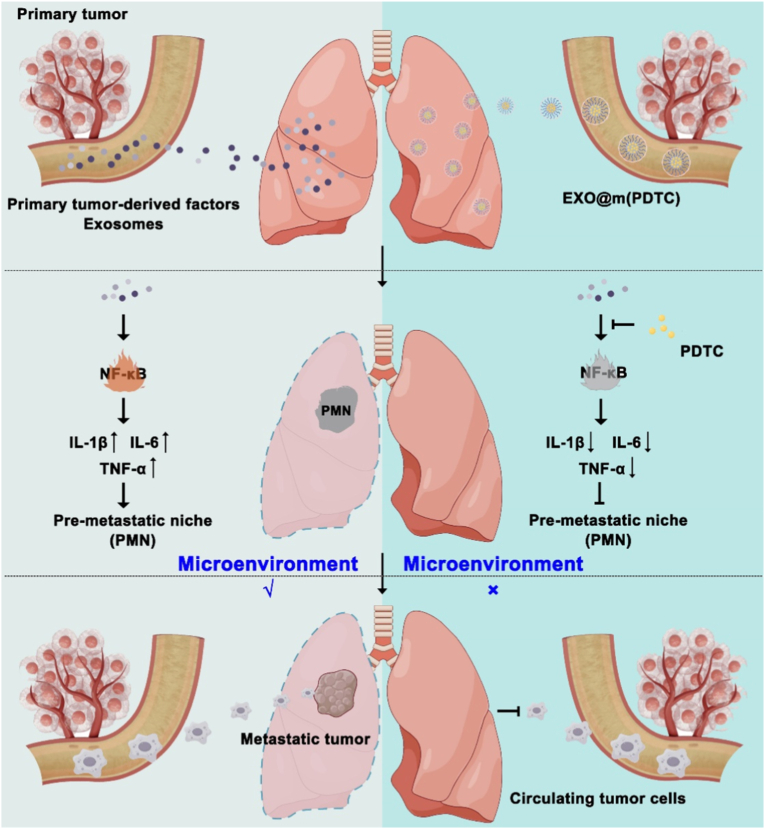
Schematic illustration of the therapeutic mechanism of EXO@m(PDTC). During breast cancer lung metastasis, the primary tumor secretes factors and exosomes that activate NF-κB signaling, triggering an inflammatory response and facilitating the formation of a PMN. This process enhances the colonization of CTCs and promotes metastatic tumor growth. EXO@m(PDTC) exhibits efficient accumulation in lung tissue, leveraging the innate lung-targeting properties of exosome membranes derived from breast cancer cells. Upon accumulation, PDTC is released to attenuate pulmonary inflammation and suppress PMN establishment. Consequently, EXO@m(PDTC) disrupts the supportive microenvironment for CTCs, leading to effective inhibition of breast cancer lung metastasis.

To address this, as shown in Scheme 1, we targeted the nuclear factor-κB (NF-κB) pathway, a master regulator of inflammation, across various pulmonary stromal cells. Studies have shown that the NF-κB pathway can mediate inflammation in various pulmonary stromal cells. For example, hypoxic tumor-derived exosomes (H-TDEs) can activate lung fibroblasts and promote lung metastasis in patients with hepatocellular carcinoma by targeting the RFX1-IL17A-p38 MAPK-NF-κB pathway [16]. Furthermore, we developed a biomimetic nanomedicine by encapsulating pyrrolidine dithiocarbamate (PDTC), an NF-κB inhibitor, within micelles coated with breast cancer-derived exosome membranes. This construct, termed EXO@m(PDTC), leverages the innate organotropism of tumor exosomes, which home preferentially to pre-metastatic sites like the lungs *via* specific integrins and caveolin-1 recognition. We show that EXO@m(PDTC) accumulates efficiently in incipient lung PMNs, inhibits NF-κB signaling in stromal cells, attenuates pulmonary inflammation, and suppresses breast cancer lung metastasis. This study provides a novel strategy for preventing metastasis through early PMN disruption and offers a promising platform for targeted nanomedicine development.

## 2 Materials and methods

### 2.1 Materials

Pluronic F-127 (PF127) and Coomassie Brilliant Blue R-250 were obtained from Beijing Solarbio Science & Technology Co., Ltd. RPMI 1640 and DMEM cell culture media were purchased from MeilunBio. Fetal Bovine Serum (FBS) was acquired from Servicebio Technology. Acrylamide (AAM), N-(3-aminopropyl)methacrylamide (APM), diallyl disulfide (DDS), tetramethylethylenediamine (TEMED), and ammonium persulfate (APS) were sourced from Shanghai Macklin Biochemical Technology Co., Ltd. Dichloromethane, dimethyl sulfoxide, triethylamine, acryloyl chloride, and diethyl ether were procured from Sinopharm Chemical Reagent Co., Ltd. DIO, DID, and the Cell counting kit (CCK-8) were supplied by Shanghai Yuanye Bio-Technology Co., Ltd. A chemiluminescence detection kit was purchased from MeilunBio.

### 2.2 Extraction of exosome membrane

The conditioned serum-free media collected from 4T1 cells after 48 h of culture were pooled for exosome isolation. Cellular debris was removed by initial centrifugation at 300×*g* for 10 min, followed by further centrifugation at 10,000×*g* for 30 min to pellet microvesicles. The pre-cleared supernatants were filtered through a 0.22 μm filter and then subjected to exosome purification using a combination of ultracentrifugation and sucrose cushion centrifugation. Specifically, the samples were centrifuged at 100,000×*g* for 90 min at 4 °C using a Sorvall™ WX 90+ ultracentrifuge equipped with a swinging bucket rotor (Thermo Fisher Scientific, USA). The resulting pellet was resuspended in 1 × PBS, layered onto 4 mL of 30% sucrose solution (prepared in 1 × PBS), and centrifuged again at 100,000×*g* for 90 min at 4 °C. The final exosome pellet was resuspended in 500 μL of 1 × PBS and stored at −80 °C until further use.

Exosomes were lysed with 1/4 × PBS (pH 8.0), and the membrane fraction was isolated by centrifugation at 2000×*g* for 5 min at 4 °C. The supernatant was discarded, and the pellet was washed with PBS, followed by repeated centrifugation to obtain purified exosome membranes. Exosome membranes were then sonicated in an FS30D bath (42 kHz, 100 W) for 10 min to enhance their dispersibility.

### 2.3 Synthesis of m(PDTC)

Acryloylated PF127 was first synthesized as follows. Briefly, PF127 (1 g) was dissolved in dichloromethane (8 mL) in a 50 mL round-bottom flask. The solution was purged with nitrogen gas and cooled to 5 °C using an ice bath. Meanwhile, trimethylamine (0.5 mL) and acryloyl chloride (0.2 mL) were dissolved in dichloromethane (8 mL) in a 25 mL flask, and this mixture was added dropwise to the PF127 solution. The reaction mixture was stirred for 12 h while maintaining the temperature at 5 °C. Subsequently, the mixture was poured into diethyl ether (100 mL) in a 500 mL beaker to precipitate the product. The precipitate was collected by filtration, and the resulting solid was dried in a vacuum oven at 50 °C. The products were analyzed using ^1^H nuclear magnetic resonance (NMR) and Fourier-transform infrared (FTIR) spectroscopy.

The acryloylated PF127 and PF127 solutions were respectively prepared by dissolving 400 mg of acryloylated PF127 or PF127 powder in 1 mL of dimethyl sulfoxide (DMSO). Simultaneously, a 100 mM PDTC solution was prepared by dissolving PDTC powder in DMSO. Subsequently, 30 μL of the PF127 solution, 10 μL of the acryloylated PF127 solution, and 10 μL of the PDTC solution were mixed and added dropwise to 950 μL of PBS solution. The mixture was thoroughly shaken after each addition and incubated on ice for 24 h.

To prepare m(PDTC), 92 μL of PBS was added to 100 μL of the above PDTC micelle solution, followed by incubation on ice for 10 min. Next, 6 μL of a cocktail solution was added to the mixture, thoroughly mixed, and incubated on ice for an additional 10 min. The cocktail solution consisted of AAM (100 mg/mL), APM (100 mg/mL), DDS (100 mg/mL), and TEMED (100 mg/mL), with a molar ratio of 10: 1: 1: 1 (AAM: APM: DDS: TEMED). Finally, 2 μL of APS (100 mg/mL) was added to the solution, mixed thoroughly, and allowed to react on ice for 2 h. The resulting solution was dialyzed overnight against PBS to obtain the final m(PDTC) solution. In addition, the above method can be followed to replace PDTC with DIO and DID, respectively, and prepare m(DIO) and m(DID). To further investigate the influence of polymerization time on the synthesis of m(PDTC), samples were collected at specific intervals following APS initiation: 0 min (before APS addition), and 5, 10, 20, 30, 45, 60, and 120 min thereafter, for subsequent analysis.

### 2.4 Preparation and characterization of EXO@m(PDTC)

The freshly obtained exosome membranes were mixed with m(PDTC) at a mass ratio of 10:1. The mixture was then subjected to sonication for 5 min using an FS30D sonicator. Subsequently, the solution was extruded 20 times sequentially through 400 nm and 200 nm polycarbonate porous membranes using a liposome extruder, followed by filtration through a 0.22 μm filter to obtain EXO@m(PDTC). The EXO@m(PDTC) solution should be prepared fresh before use, and freezing should be avoided whenever possible. Furthermore, EXO@m(DIO) and EXO@m(DID) can be prepared according to the above method.

Size and zeta potential measurements were conducted using a Zetasizer Nano ZS instrument (Malvern Panalytical, UK) at room temperature. The EXO@m(PDTC) sample concentration for these measurements was adjusted to 0.1 mg/mL (based on PDTC content). In addition, the stability of the nanoparticles in PBS or DMEM supplemented with 10% FBS was evaluated over 7 days by monitoring the change in particle size. Morphological characterization was performed using high-resolution transmission electron microscopy (TEM, JEM-2100F, JEOL, Japan). For TEM imaging, 50 μL of EXO@m(PDTC) solution was applied to copper grids, air-dried for 10 min, negatively stained with 2% phosphotungstic acid for 2 min, and subsequently examined.

For FTIR spectroscopy analysis, samples (0.1 mg/mL) were lyophilized into powder, mixed with KBr, and pressed into pellets. Chemical composition was analyzed using an FTIR spectrometer (Nicolet iS50, Thermo Fisher Scientific, USA). Spectra were recorded in transmission mode from 4000 to 400 cm^−1^ at room temperature with a resolution of 0.5 cm^−1^. Additionally, ultraviolet–visible (UV–Vis) absorption of the sample solutions (0.1 mg/mL) was measured using a spectrophotometer (UH4150, Hitachi, Japan) across a wavelength range of 200–700 nm.

To assess whether the preparation process of EXO@m(PDTC) affects exosomal membrane components, sodium dodecyl sulfate-polyacrylamide gel electrophoresis (SDS-PAGE) was conducted. Briefly, 10 μL of each sample (0.1 mg/mL) was resuspended in 2 × loading buffer. After incubation at 70 °C for 15 min, the samples were loaded onto a 10% (w/v) polyacrylamide gel and subjected to electrophoresis at 120 V for 60 min. Subsequently, the gel was stained with Coomassie Brilliant Blue R-250 for 1 h at room temperature. This was followed by overnight destaining in a solution containing 10% glacial acetic acid and 30% methanol. Finally, the gel was photographed to document the distribution of protein bands.

To test the drug release profiles of PDTC from EXO@m(PDTC), 1 mL of each sample suspension was separately incubated in PBS with or without 1 mM dithiothreitol (DTT) at 37 °C for 2 h to allow complete hydrolysis. Subsequently, the mixture was quantitatively transferred into a dialysis bag (molecular weight cut-off: 3.5 kDa). The bag was immersed in a 50 mL centrifuge tube containing 40 mL of PBS and continuously shaken at 100 rpm in a constant-temperature shaker. At predetermined time points (0.5, 1, 2, 4, 8, 16, 24, 48, and 72 h), 1 mL aliquots were withdrawn from the external PBS medium, with an equal volume of pre-warmed PBS replenished each time. The absorbance of each sample was measured using a UV–Vis spectrophotometer, and the cumulative release rate was calculated based on a standard curve.

### 2.5 Biocompatibility assay

The cytotoxicity of m(PDTC) and EXO@m(PDTC) was evaluated in BEAS-2B, L929, and RAW264.7 cell lines using a CCK-8 assay. Cell suspensions (200 μL/well) containing 5000 cells were seeded into 96-well plates and incubated at 37 °C for 24 h. Subsequently, the cells were treated with varying concentrations of m(PDTC) and EXO@m(PDTC) (based on PDTC concentration) for an additional 24 h. After treatment, 10 μL of CCK-8 solution was added to each well, followed by a 30-min incubation. Absorbance was then measured at 450 nm using a microplate reader.

To evaluate the hemolytic activity of m(PDTC) and EXO@m(PDTC), fresh heparin-anticoagulated mouse blood was collected, and 4 mL of whole blood was mixed with 8 mL of saline. Red blood cells (RBCs) were isolated by centrifugation at 1000×*g* for 15 min and washed five times with sterile saline. After the final wash, the RBCs were resuspended in 40 mL of saline. Subsequently, 0.2 mL of the diluted RBC suspension was mixed with 0.8 mL of m(PDTC) or EXO@m(PDTC) to achieve final concentrations of 50, 100, and 150 μM. The mixture was vortexed briefly and incubated at room temperature for 4 h. Following incubation, the samples were vortexed again and centrifuged at 1000×*g* for 10 min. The supernatant (400 μL) was analyzed using UV–vis absorbance spectroscopy. Negative and positive controls were prepared by incubating 0.2 mL of the diluted RBC suspension with 0.8 mL of saline and 0.8 mL of distilled water, respectively.

### 2.6 Cellular uptake assay

To visualize cellular uptake, EXO@m(DIO) was prepared and incubated with BEAS-2B, L929, and RAW264.7 cell lines. Briefly, cells were cultured on polylysine-coated glass coverslips in a 24-well plate. After reaching confluence, the cells were treated with m(DIO) and EXO@m(DIO) at concentrations of 10 μM, 50 μM, and 100 μM, respectively, for 8 h. Subsequently, the cells were washed three times with PBS, fixed with paraformaldehyde and 0.5% Triton X-100, and mounted on slides using DAPI-containing fluorescent mounting medium. Cellular uptake was observed under a confocal microscope (TCS-SP8, Leica, Germany) under consistent imaging parameters for all experiments. The fluorescence intensity was quantified using the ImageJ software.

To elucidate the specific endocytic mechanism of EXO@m(DIO), cells were pretreated with inhibitors targeting cell surface receptors, and subsequent changes in intracellular uptake efficiency were assessed. Specifically, three common endocytic receptor inhibitors were selected: erlotinib, an EGFR inhibitor for BEAS-2B cells; cilengitide, an αvβ3 integrin receptor inhibitor for L929 cells; and BLT-1, a scavenger receptor inhibitor for RAW264.7 cells. Each inhibitor was applied at a concentration of 10 μM to the respective cell lines cultured in six-well plates, followed by incubation for 2 or 12 h. Subsequently, the cells were treated with 100 μM of either m(DIO) or EXO@m(DIO) for 8 h. Cellular uptake was visualized using a confocal microscope under consistent imaging parameters, and fluorescence intensity was quantified with ImageJ software.

### 2.7 Western blot

To evaluate the therapeutic efficacy of EXO@m(PDTC), we examined alterations in proteins associated with the NF-κB signaling pathway. BEAS-2B, L929, and RAW264.7 cells were divided into six groups. Four groups were treated with 50% 4T1 TCM (a 1:1 mixture of tumor-conditioned medium and normal medium) for 6 h. One group was treated with lipopolysaccharide (LPS, 1 μM) as a positive control, and an untreated group served as the negative control. Subsequently, experimental groups were incubated with PBS, PDTC (100 μM), m(PDTC) (100 μM), or EXO@m(PDTC) (100 μM) for 24 h. Proteins were extracted from BEAS-2B, L929, and RAW264.7 cells using radioimmunoprecipitation assay buffer. Equal protein quantities were separated by 10% SDS-PAGE and transferred onto polyvinylidene fluoride (PVDF) membranes (Millipore Corp., USA). The membranes were blocked with 3% bovine serum albumin (BSA; Solarbio, China) at 37 °C for 1 h, followed by overnight incubation with primary antibodies (NF-κB and p-NF-κB, Zenbio, China; IκBα and p-IκBα, Boster, China) at 4 °C. Afterward, the membranes were incubated with secondary antibodies (Epizyme Biotech, China) at 37 °C for 1 h. Protein bands were visualized using a chemiluminescence detection kit with the FUSION SOLO 4S WL imager system (Vilber, France), and band densities were quantified using ImageJ.

### 2.8 RNA isolation and quantitative reverse transcription PCR (qRT-PCR)

BEAS-2B, L929, and RAW264.7 cells were treated as described above. Total RNA was extracted using the Direct-zol RNA Miniprep Plus Kit (Zymo Research, USA). Reverse transcription was performed with the High-Capacity cDNA Reverse Transcription Kit (Thermo Fisher Scientific, USA). Target-specific primers (IL-1β, IL-6, and TNF-α) were selected for detection. qPCR was conducted using PowerUp SYBR™ Green PCR Master Mix (Thermo Fisher Scientific, USA), with miRNA expression levels normalized to GAPDH according to the manufacturer's protocol. Relative gene expression was calculated using the comparative cycle threshold method (2-ΔΔCt), normalized to the housekeeping gene Rps18. Primer sequences are provided in Table S1.

### 2.9 The *in vivo* pharmacokinetics studies

To evaluate the *in vivo* pharmacokinetics, DID was employed as a surrogate for PDTC. Healthy female BALB/c mice (18–20 g; Jinan HFK Bioscience Co., Ltd., China) were allocated into three groups: free DID, m(DID), and EXO@m(DID). Each group received an intravenous injection of DID at a dose of 0.25 mg/kg *via* the tail vein. Blood samples were collected from the tail vein at predetermined time points: 0.5, 1, 2, 4, 8, 12, 24, and 48 h post-injection. The fluorescence intensity of DID in the blood was quantified using an IVIS imaging system.

### 2.10 Construction of lung PMN induced by TCM

4T1 cells were seeded in 10 cm^2^ culture dishes and cultured in 10 mL of complete RPMI 1640 medium. When the cells reached 90% confluence, the supernatant was collected and centrifuged at 1000×*g* for 5 min. The resulting supernatant was then filtered through a 0.22 μm membrane to obtain 4T1 TCM. BALB/c female mice (18–20 g, Jinan HFK Bioscience Co., Ltd., China) received 200 μL of 4T1 TCM *via* tail vein injection every other day for a total of four administrations. All experimental procedures were conducted in accordance with the Animal Experimentation Guidelines of Qingdao University (Qingdao, Shandong, China) and were approved by the Ethics Committee of the Medical College of Qingdao University.

To confirm the construction of the PMN model, immunohistochemical staining of lung tissue was conducted. Paraffin-embedded sections were dewaxed and rehydrated. Endogenous peroxidase activity was quenched using 3% H_2_O_2_, and antigen retrieval was performed by heating the sections in citrate buffer for 10 min. The sections were then blocked with 5% normal goat serum for 1 h at room temperature, followed by overnight incubation at 4 °C with primary antibodies against CCL-2, S100A8, and IL-1β. Subsequently, the samples were incubated with biotin-conjugated secondary antibodies. Staining signals were detected using an avidin-biotin complex peroxidase system. Positive antibody binding was visualized with a peroxidase substrate kit, specifically 3,3′-diaminobenzidine (DAB). Nuclei were counterstained with hematoxylin. Quantitative analysis of immunohistochemical staining was performed using ImageJ.

### 2.11 Measurement of the targeting ability for lung PMN induced by TCM

Female BALB/c mice were used to establish a 4T1 breast cancer lung PMN model using the aforementioned method. Two days after the final injection of 4T1 TCM, the 12 mice were randomly allocated into two groups: one group received an intravenous injection of 200 μL m(DID), while the other received 200 μL EXO@m(DID), with a DID concentration of 0.1 mg/mL. Additionally, control groups of normal mice were injected with PBS or EXO@m(DID), respectively. Twenty-four hours post-injection, whole-animal imaging was conducted using the IVIS Spectrum imaging system. The mice were then euthanized, and their lungs and other major organs were harvested, rinsed with PBS, and placed in a dish. Fluorescence imaging results and average radiant intensities were recorded using the IVIS Spectrum system. Moreover, three lung samples were embedded in optimal cutting temperature (OCT) compound, rapidly frozen at −20 °C for 24 h, and sectioned into 8 μm slices. The frozen sections were stained with DAPI and examined under a fluorescence microscope.

### 2.12 The determination of EXO@m(PDTC)'s anti-metastasis effect

Thirty-six female BALB/c mice were randomly divided into six groups, four of which received both TCM treatment and drug intervention. Specifically, 200 μL of 4T1 TCM was intravenously administered *via* tail vein injection on days 1, 3, 5, and 7 (totaling four administrations). On alternating days (days 2, 4, 6, and 8), the mice received 200 μL of PBS, PDTC, m(PDTC), or EXO@m(PDTC) *via* tail vein injection, with PDTC administered at a dose of 40 mg/kg. Two days after the final drug injection, all mice were intravenously inoculated with 50,000 4T1-Luc cells. Control groups comprised mice injected with either PBS or 4T1-Luc cells alone on day 10. Pulmonary tumor nodule formation was evaluated on days 17, 24, and 31 using the IVIS Spectrum imaging system. Subsequently, the mice were euthanized, and their lungs and other major organs were harvested, rinsed with PBS, and placed in a dish. Bioluminescence imaging was performed using the IVIS Spectrum system. Additionally, three lung samples were fixed in 4% paraformaldehyde for 24 h, embedded in paraffin (Thermo Fisher Scientific, USA), and incubated at 60 °C for 12 h. Sections of 6 μm thickness were prepared using a paraffin microtome (Bio-Rad, USA) and subjected to hematoxylin and eosin (HE) staining. Tissue images were acquired using a microscope (Nikon, Japan).

### 2.13 Measurement of the targeting ability for lung PMN induced by the orthotopic 4T1 tumor

To establish the 4T1 breast cancer *in situ* model, female BALB/c mice were injected with 4T1-Luc cells (1 × 10^5^) into the mammary fat pad. The method for detecting PMN formation follows the procedure detailed in Section 2.10. Two weeks post-inoculation, the 18 tumor-bearing mice were randomly divided into three groups: (1) a control group injected with PBS, (2) a group receiving 200 μL of m(DID) intravenously, and (3) a group administered 200 μL of EXO@m(DID) (DID concentration: 0.2 mg/mL). An additional control group of healthy mice was injected with EXO@m(DID). Forty-eight hours post-injection, whole-body imaging was performed using the IVIS Spectrum system. Subsequently, the mice were euthanized, and their lungs and major organs were harvested, rinsed with PBS, and placed in a dish. Fluorescence imaging and average radiant intensity measurements were obtained using the IVIS Spectrum system. Furthermore, three lung samples were embedded in OCT compound, flash-frozen at −20 °C for 24 h, and sectioned into 8 μm slices. The frozen sections were stained with DAPI and visualized under a fluorescence microscope.

### 2.14 Evaluation of EXO@m(PDTC)'s effect on preventing lung metastasis of orthotopic breast cancer

Thirty female BALB/c mice were randomly allocated into six groups, five of which were used to establish an orthotopic 4T1 breast cancer model and underwent drug intervention. 4T1-Luc cells (1 × 10^5^) were injected into the mammary fat pad of each mouse. On days 2, 4, 6, and 8 post-injection, the mice received 200 μL of PBS, PDTC, m(PDTC), or EXO@m(PDTC) *via* tail vein injection, with PDTC administered at a dose of 40 mg/kg. Normal mice served as controls. Body weight and tumor volume were monitored throughout the study. Pulmonary metastasis was assessed on days 23 and 37 using the IVIS Spectrum imaging system. Subsequently, the mice were euthanized, and their lungs and other major organs were harvested, rinsed with PBS, and placed in a Petri dish. Bioluminescence imaging was performed using the IVIS Spectrum system. Additionally, three lung samples were fixed in 4% paraformaldehyde for 24 h, embedded in paraffin, and incubated at 60 °C for 12 h. Sections of 6 μm thickness were prepared using a microtome and subjected to HE staining. Tissue images were acquired using a light microscope.

In addition, to measure the biosafety of EXO@m(PDTC), histopathological evaluation of other major organ samples, including heart, liver, spleen, and kidney, was performed. These organs were fixed in 4% paraformaldehyde for 24 h, embedded in paraffin, and incubated at 60 °C for 12 h. Sections of 6 μm thickness were prepared using a microtome and subjected to HE staining. Tissue images were acquired using a light microscope. Furthermore, immediately after euthanizing the mice, blood was collected using an anticoagulation tube containing EDTA2K. And the serum was extracted by centrifugation at 1000*g* for 30 min. The relevant expression of IL-1β, IL-6, TNF-α, RBC, leukocyte (WBC), platelet (PLT), creatinine (CRE), aspartate transaminase (AST), alanine transaminase (ALT), high-density lipoprotein (HDL), and triglycerides (TG) was detected.

### 2.15 mRNA-Seq data analysis

An orthotopic breast cancer model was constructed using the aforementioned method and subsequently treated with either PBS or EXO@m(PDTC). Following treatment, lung tissues were harvested from three mice per group for transcriptome sequencing. Experiments performed by Genefund Biotech (Shanghai, China), and the sequencing protocol was as follows:

Total RNA was extracted from lung tissues using TRIzol reagent (Thermo Fisher Scientific, USA), followed by DNase treatment to eliminate genomic DNA contamination. mRNA was isolated using the KAPA Stranded mRNA-Seq Kit (Roche Diagnostics GmbH, Germany), and RNA-Seq libraries were prepared with the same kit for Illumina platforms. Sequencing was performed on an Illumina system in paired-end mode (2 × 150 bp).

Raw sequencing data underwent quality control and alignment, after which gene expression levels were quantified as FPKM (fragments per kilobase of exon per million fragments mapped) using StringTie v1.3.4d (parameters: e--rf). Differential gene expression analysis was conducted with edgeR v3.24.2, with adjusted P-values calculated *via* the false discovery rate (FDR) method to account for multiple testing. Genes with an adjusted P < 0.05 and |log2FC| ≥ 1 were considered statistically significant for downstream analysis. Gene annotations were obtained from the Ensembl genome browser 96 database (http://www.ensembl.org/index.html), and functional enrichment analysis (GO and KEGG) was performed using ClusterProfiler v3.4.4.

### 2.16 Statistical analysis

All statistical analysis was performed using GraphPad Prism 10.0 software, repeated at least three times. All quantitative data are presented as the mean ± SD. The significance between the two groups was evaluated using the Student's t-test, and multi-group comparisons were performed using one-way and two-way analysis of variance (ANOVA). A p-value of less than 0.05 was deemed statistically significant, indicating meaningful differences in the observed data. The minimum level of significance was set at *p < 0.05, **p < 0.01, ***p < 0.001, and ****p < 0.0001.

## 3 Results

### 3.1 Preparation and characterization of m(PDTC) and EXO@m(PDTC)

**Fig. 1:**
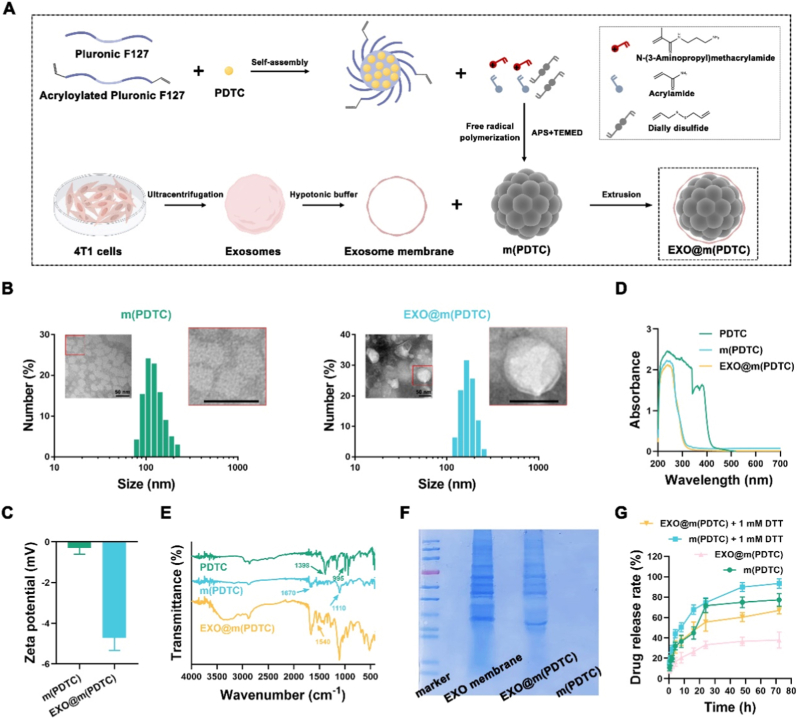
Synthesis and characterization of EXO@m(PDTC). (A) Schematic of the fabrication process of EXO@m(PDTC); (B) Hydrodynamic size distribution and representative TEM images of m(PDTC) and EXO@m(PDTC), scale bar = 50 nm; (C) Zeta potential measurements of m(PDTC) and EXO@m(PDTC); (D) The UV–Vis absorption spectrum of m(PDTC) and EXO@m(PDTC); (E) The FTIR spectra of m(PDTC) and EXO@m(PDTC); (F) Protein composition analysis of m(PDTC), EXO membrane, and EXO@m(PDTC) *via* SDS-PAGE with Coomassie brilliant blue staining; (G) *In vitro* release profiles of PDTC from m(PDTC) and EXO@m(PDTC) in PBS with or without 1 mM DTT.

PF127, a nonionic triblock copolymer of poly(ethylene oxide)-poly(propylene oxide)-poly(ethylene oxide), possesses the ability to self-assemble into micelles and encapsulate PDTC [17]. To enhance micellar stability, we chemically modified PF127 to synthesize acryloylated PF127, which facilitates the formation of micelles with surface-exposed double bonds. To verify the successful synthesis of acryloylated PF127, ^1^H NMR and FTIR spectroscopy analyses were conducted. The ^1^H NMR results revealed three peaks between 5.9 and 6.4 ppm, corresponding to the acrylate protons at the termini of acryloylated PF127 (Fig. S1). The FTIR spectra indicated a significant reduction in the hydroxyl stretching vibration peak at 2880 cm^−1^ and the emergence of a carbonyl stretching vibration at 1720 cm^−1^ (Fig. S2). These findings confirm that PF127 forms an ester bond with acryloyl chloride *via* hydroxyl groups, demonstrating the successful synthesis of acryloylated PF127. Subsequently, through free radical polymerization, a cross-linked polymer network was formed on the micellar surface (Fig. 1A), yielding the structure designated as m(PDTC). The drug loading rate of m(PDTC) was determined to be 15.23%–19.05%. The monomeric constituents of this surface network include AAM, APM, and DDS. APM confers a nearly neutral surface potential to m(PDTC), thereby promoting exosome membrane encapsulation, while DDS confers intracellular degradability due to its responsiveness to redox conditions. Collectively, the m(PDTC) system not only enhances micellar stability but also improves exosome membrane encapsulation efficiency.

It should be noted that the incubation duration significantly influences the formation of m(PDTC), as the polymerization reaction requires sufficient time to proceed. To assess this, dynamic light scattering (DLS) was employed to monitor changes in the size and zeta potential of m(PDTC) over various polymerization intervals. The corresponding results are presented in Fig. S3. The experimental data indicate that during the initial 30 min of incubation, the size of m(PDTC) increased progressively with prolonged incubation time. Subsequently, after this period, the particle size stabilized. Throughout the polymerization process, the zeta potential of m(PDTC) showed no significant fluctuations, a phenomenon that may be attributed to the inherent properties of the polymerizing monomers. Additionally, to avoid interference with the free radical polymerization, potential inhibitors were carefully excluded. For example, polymerization inhibitors were removed from the monomers, and oxygen was eliminated to prevent its inhibitory effect. To further control the reaction, the quantities of initiators and catalysts, as well as the polymerization temperature, were rigorously regulated to avoid an excessively vigorous polymerization reaction.

Exosomes were isolated from the serum-free culture medium of 4T1 cells *via* ultracentrifugation, and their membranes (EXO membranes) were subsequently extracted using a hypotonic solution. These membranes were mixed with m(PDTC) and extruded through polycarbonate membranes to produce EXO@m(PDTC). To determine the optimal EXO membrane concentration for effective m(PDTC) encapsulation, two complementary methods were employed. First, exploiting the size difference between EXO@m(PDTC) and free m(PDTC), m(PDTC) (5 μg/mL) was incubated with varying concentrations of EXO membranes (0–80 μg/mL), followed by hydrodynamic size measurement *via* DLS. Second, a fluorescence quenching assay was conducted using FITC-labeled m(PDTC). After incubating the labeled nanoparticles with the aforementioned membrane concentrations, the initial fluorescence intensity was measured. Subsequently, 1 μg/mL of FITC antibody was added, and fluorescence quenching was assessed following a 30-min incubation. As illustrated in Fig. S4, membrane concentrations below 50 μg/mL resulted in a progressive increase in particle size and a concurrent decrease in fluorescence quenching efficiency. However, at concentrations of 50 μg/mL and above, both particle size and quenching efficiency stabilized, indicating that complete encapsulation of m(PDTC) is achieved at a 10:1 mass ratio (membrane to nanoparticle). Consequently, this optimized ratio was adopted for all subsequent experiments.

DLS revealed size distributions of 78.82–220.20 nm for m(PDTC) and 122.40–255.00 nm for EXO@m(PDTC) (Fig. 1B). Transmission electron microscopy (TEM) confirmed that the exosomal membrane coating increased nanoparticle size while maintaining the spherical morphology of m(PDTC). A distinct membrane bilayer was observed surrounding EXO@m(PDTC). Zeta potential measurements (Fig. 1C) demonstrated that EXO@m(PDTC) exhibited greater negative surface charge than m(PDTC), attributable to the inherent electronegativity of exosomal membranes [18]. This finding corroborates successful membrane coating and aligns with known characteristics of exosomal surfaces, which are rich in negatively charged phospholipids and membrane proteins [19]. The smaller hydrodynamic diameter and anionic surface charge of EXO@m(PDTC) promote evasion of reticuloendothelial system clearance, thereby enhancing PDTC delivery efficiency *in vivo*.

The UV–Vis absorption spectrum revealed that PDTC exhibits strong absorption in the 300–400 nm range, whereas m(PDTC) and EXO@m(PDTC) display negligible absorption in this region, confirming the effective encapsulation of PDTC (Fig. 1D). FTIR spectroscopy further demonstrated characteristic peaks for PDTC at 1398 cm^−1^ (N-CSS stretching vibration) and 995 cm^−1^ (C=S stretching vibration) (Fig. 1E). In contrast, these peaks were absent in the m(PDTC) spectrum, which instead exhibited peaks at 1670 cm^−1^ (C=C stretching vibration) and 1110 cm^−1^ (C-C-O symmetric stretching vibration of PF127), indicating the formation of a polymeric network. Notably, the FTIR spectrum of EXO@m(PDTC) showed a distinct peak at 1540 cm^−1^ (amide II, attributed to N-H bending vibrations of peptide groups), confirming successful exosome membrane coating on m(PDTC) [20].

The proteins on exosome membranes are key components that confer targeting capabilities. For instance, caveolin-1 (Cav-1), present on the membrane surface of breast cancer cell-derived exosomes, mediates their targeted delivery to lung tissue [21]. To examine whether the preparation process of EXO@m(PDTC) alters exosomal membrane protein composition, we analyzed protein profiles using SDS-PAGE with Coomassie brilliant blue staining. As shown in Fig. 1F, no detectable protein bands were observed in the m(PDTC) group, confirming the absence of Coomassie brilliant blue-binding substances. Notably, EXO@m(PDTC) exhibited protein bands comparable to those of EXO membrane, demonstrating preserved 4T1 cell exosome membrane composition and suggesting retained lung-targeting potential.

To characterize drug release kinetics, we monitored PDTC release from EXO@m(PDTC) and m(PDTC) over 72 h (Fig. 1G). Both formulations displayed sustained-release profiles, with EXO@m(PDTC) exhibiting superior release prolongation, likely attributable to the relative stability of m(PDTC) and delayed PDTC diffusion mediated by exosome membrane encapsulation. Furthermore, DTT significantly enhances the drug release efficiency of both m(PDTC) and EXO@m(PDTC), indicating their redox-responsive release capability. However, the cumulative release of EXO@m(PDTC) remains lower than that of m(PDTC), suggesting that the exosome membrane improves micelle stability. This prolonged drug retention at target sites is particularly advantageous for therapies requiring sustained PMN inhibition.

The stability of EXO@m(PDTC) was evaluated (Fig. S5). The results demonstrate that both m(PDTC) and EXO@m(PDTC) maintained consistent particle sizes in PBS at 4 °C over 7 days, confirming their stability under low-temperature storage conditions. Under simulated physiological conditions at 37 °C, a slight increase in particle size was observed for both formulations, likely resulting from interactions with serum proteins. Despite this, the size change remained minimal throughout the 7 days, indicating sufficient stability for physiological applications and supporting their potential for *in vivo* use. It should be pointed out that, however, when the incubation period was extended to 14 days under identical conditions, both formulations exhibited partial precipitation, possibly due to aggregation caused by serum protein denaturation. Considering the typical *in vivo* circulation duration of the nanomedicine, we conclude that its stability is adequate for effective functionality during application.

In conclusion, EXO@m(PDTC) can be efficiently fabricated by coating 4T1 cell-derived exosomal membranes onto m(PDTC). This hybrid construct retains the membrane components of natural exosomes, endowing it with lung-targeting potential. Moreover, EXO@m(PDTC) may enhance the pharmacokinetic profile of PDTC through its redox-responsive properties and the exosomal membrane-mediated modulation of PDTC diffusion.

### 3.2 Cell uptake, cytotoxicity, and blood compatibility

**Fig. 2:**
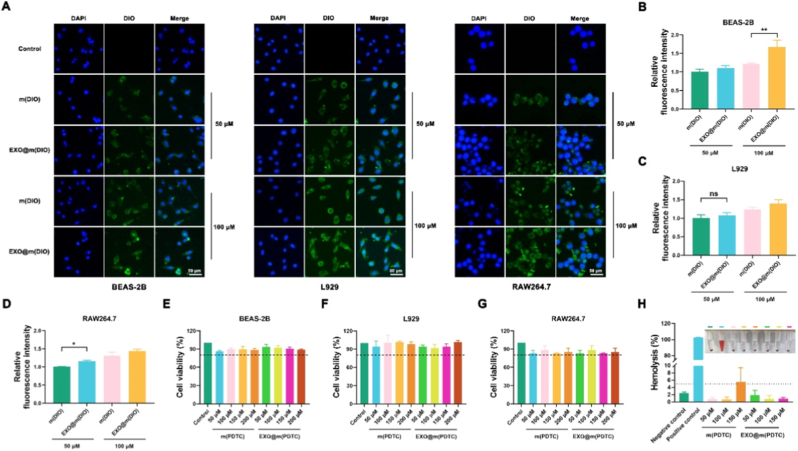
Cellular uptake and biocompatibility evaluation of nanomedicines. (A) Intracellular distribution of m(DIO) and EXO@m(DIO) in BEAS-2B, L929, and RAW264.7 cells following 8-h incubation; (B–D) Quantitative analysis of fluorescence intensity indicating the uptake of m(DIO) and EXO@m(DIO) in BEAS-2B, L929, and RAW264.7 cells; (E–G) Viability of BEAS-2B, L929, and RAW264.7 cells treated with m(PDTC) and EXO@m(PDTC) at concentrations ranging from 50 to 200 μM for 48 h; (H) Hemolysis assay of m(PDTC) and EXO@m(PDTC) at concentrations of 50, 100, and 150 μM.

To evaluate the endocytosis efficiency of EXO@m(PDTC), m(DIO), and EXO@m(DIO) were prepared using the fluorescent dye DIO as a substitute for PDTC. Since our objective was to modulate inflammatory responses in diverse pulmonary stromal cells, BEAS-2B, L929, and RAW264.7 cells were incubated with m(DIO) and EXO@m(DIO) at concentrations of 50 μM and 100 μM for 8 h. As illustrated in Fig. 2A, all three cell types exhibited efficient uptake of both m(DIO) and EXO@m(DIO), demonstrating a concentration-dependent pattern. At equivalent concentrations, EXO@m(DIO) showed higher cellular uptake efficiency than m(DIO), as further confirmed by quantitative analysis (Fig. 2B–D). This phenomenon can be attributed to the presence of proteins, lipids, and sugars on the exosome membrane surface, which enhance nanomedicine-cell interactions and thus improve endocytosis efficiency [22]. Notably, for L929 and RAW264.7 cells at 100 μM, no significant difference in uptake efficiency was observed between m(DIO) and EXO@m(DIO), likely due to cellular saturation at this concentration. To clarify whether the lack of significant difference in cellular uptake between m(DIO) and EXO@m(DIO) at 100 μM was due to saturation effects, we tested two concentration gradients (10 and 100 μM). As shown in Figs. S6 and S7, the uptake for both m(DIO) and EXO@m(DIO) approached 100% at 100 μM, with no statistical difference. In contrast, at 10 μM, EXO@m(DIO) demonstrated significantly higher uptake efficiency than m(DIO). These results indicate that at high concentrations, uptake saturation masks the inherent difference between the two carriers. At lower, non-saturating concentrations, the exosome membrane coating confers a clear advantage, leading to superior cellular uptake of EXO@m(DIO). In this study, m(DIO) and EXO@m(DIO) served as proxies to assess endocytosis efficiency, with results being generalizable to m(PDTC) and EXO@m(PDTC). This is because the encapsulated drugs minimally influence the size and surface physicochemical properties of the nanomedicines, thereby preserving their endocytic characteristics.

To elucidate the specific endocytic mechanism of EXO@m(PDTC), we selected BEAS-2B cells treated with the EGFR inhibitor erlotinib, L929 cells treated with the integrin inhibitor cilengitide, and RAW264.7 cells treated with the scavenger receptor inhibitor BLT-1. These inhibitors were applied to their respective cell lines, and changes in the internalization of EXO@m(DIO) before and after treatment were observed. The results demonstrated that treatment with each inhibitor reduced the endocytic efficiency of EXO@m(DIO) to varying degrees (Fig. S8), suggesting that EGFR, integrins, and scavenger receptors play significant roles in the receptor-mediated endocytosis of EXO@m(PDTC) in BEAS-2B, L929, and RAW264.7 cells, respectively. However, endocytosis was not completely suppressed by any inhibitor, indicating that exosome membrane-mediated endocytosis is not reliant on a single receptor but may involve multiple receptors simultaneously. For a given cell type, endocytosis may be predominantly mediated by a specific receptor. Additionally, the lipid bilayer of the exosome membrane contributes substantially to enhancing endocytic efficiency. In conclusion, the endocytic mechanisms of EXO@m(PDTC) appear to vary across cell types, potentially involving multiple receptors and the influence of lipid components.

To assess the cytotoxicity of the nanomedicines, three cell types were treated with varying concentrations of m(PDTC) and EXO@m(PDTC). The CCK-8 assay results indicated that both compounds exhibited low cytotoxicity, with cell viability exceeding 80% across all cell types, even at a concentration of 200 μM after 24 h of treatment (Fig. 2E–G). Furthermore, hemolysis assays demonstrated that EXO@m(PDTC) maintained hemolysis rates below 5% at concentrations of 50 μM, 100 μM, and 150 μM (Fig. 2H). In contrast, m(PDTC) showed a slight hemolytic effect at 150 μM. These results suggest that exosome membrane encapsulation may improve the hemocompatibility of m(PDTC) by altering its surface physicochemical properties. Together, the *in vitro* safety assessments, including evaluations of cytotoxicity and hemocompatibility, revealed negligible risk to tissue cells and erythrocytes, indicating favorable biosafety and hemocompatibility of the nanomaterial.

### 3.3 *In vitro* anti-inflammatory effect of EXO@m(PDTC)

**Fig. 3:**
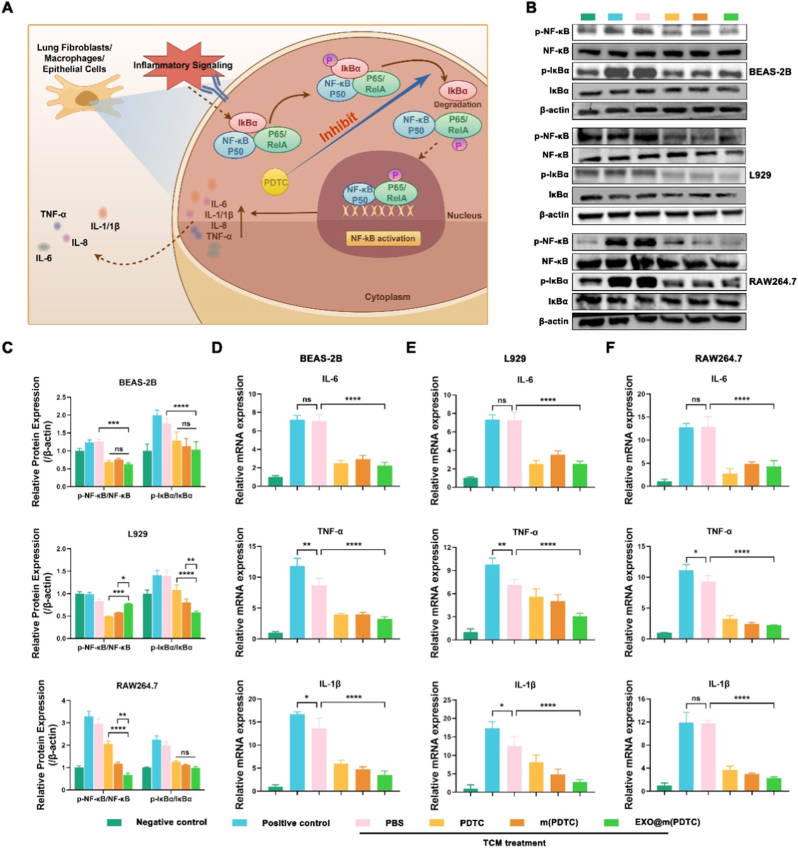
Evaluation of the anti-inflammatory effect of EXO@m(PDTC). (A) Schematic representation of the mechanism by which PDTC inhibits the NF-κB signaling pathway; (B) Western blot analysis of phosphorylated NF-κB (p-NF-κB), NF-κB, phosphorylated IκBα (p-IκBα), and IκBα expression in BEAS-2B, L929, and RAW264.7 cells following treatment with the nanomedicines; (C) Quantification of protein expression levels from Western blot analysis using ImageJ software; (D–F) mRNA expression levels of IL-1β, IL-6, and TNF-α measured by qRT-PCR in BEAS-2B, L929, and RAW264.7 cells after nanomedicine treatment.

The NF-κB pathway serves as a master regulator of inflammation and contributes to the formation of PMN in lung tissue by modulating stromal cells (Fig. 3A) [23]. Canonical NF-κB signaling directly promotes the production of proinflammatory cytokines, including TNF-α and IL-6, whereas IL-1β synthesis is regulated through a two-step process involving transcription and maturation [24]. The central event in canonical NF-κB activation involves signal-induced phosphorylation of IκB molecules by IKKs [25]. Under steady-state conditions, RelA and p50 remain sequestered in the cytoplasm by IκB. Upon phosphorylation, IκB dissociates from these nuclear factors, enabling their translocation into the nucleus to drive inflammatory gene expression. PDTC can suppress NF-κB activation by functioning as an antioxidant that inhibits the ubiquitination and subsequent proteasomal degradation of IκBα, its inhibitory protein [26]. This mechanism prevents NF-κB translocation to the nucleus.

To evaluate the anti-inflammatory effects of various treatments, we stimulated normal lung stromal cells with 4T1 tumor-conditioned medium (TCM) and administered PDTC, m(PDTC), or EXO@m(PDTC) to assess their inhibitory effects on cytokine-induced inflammation. Western blot analysis and relative quantification demonstrated that nanomedicine treatment reduced the levels of phosphorylated NF-κB and IκBα (Fig. 3B and C). However, EXO@m(PDTC) did not show a significant advantage; in some cases, its efficacy was even lower than that of PDTC or m(PDTC). For instance, in L929 cells, EXO@m(PDTC) exhibited weaker inhibition of NF-κB phosphorylation compared to both PDTC and m(PDTC). We hypothesize that these results may be explained by two factors: (1) *In vitro*, although EXO@m(PDTC) may have higher cellular uptake efficiency than m(PDTC), its drug release rate could be slower, thereby reducing its inhibitory effect; (2) Protein phosphorylation involves spatiotemporal dynamics, and differences in the drug release profiles between EXO@m(PDTC) and m(PDTC) may lead to variations in phosphorylation inhibition. Additionally, quantitative reverse transcription polymerase chain reaction (qRT-PCR) results demonstrated that drug treatment significantly downregulated IL-1β, IL-6, and TNF-α expression (Fig. 3D–F). In summary, our findings suggest that EXO@m(PDTC) holds only a comparative, rather than an absolute, advantage *in vitro*, likely due to suboptimal drug release kinetics. Nevertheless, we anticipate that its *in vivo* performance may offset this limitation.

### 3.4 The targeting ability of EXO@m(PDTC) for lung PMN induced by TCM

We first evaluate the pharmacokinetic profile of EXO@m(PDTC) in healthy mice. The results, presented in Fig. S9, demonstrate that the circulation time of m(DID) was markedly prolonged compared to the hydrophobic fluorescent dye DID. This enhancement is primarily attributable to the introduction of chemical cross-linking, which substantially improved micellar stability. Furthermore, EXO@m(DID) exhibited an even longer circulation duration than m(DID), an effect ascribed to the incorporation of the exosome membrane. Notably, m(DID) reached its peak concentration 1 h post-administration, whereas EXO@m(DID) achieved its maximum at 2 h. This temporal disparity further underscores the influence of the exosome membrane on the pharmacokinetic behavior of the nanomedicine.

To assess the targeting capability of EXO@m(PDTC) toward pulmonary PMNs, we first validated the formation of PMNs in lung tissues induced by tail vein injection of TCM. As illustrated in Fig. S10, varying volumes of 50% TCM (a 1:1 mixture of TCM and normal medium) consistently induced the expression of CCL-2 and S100A8, two PMN marker proteins [27]. Notably, PMNs induced by 200 μL TCM exhibited greater maturity. Meanwhile, the expression levels of the inflammatory cytokine IL-1β in murine lung tissues exhibited significant variations across experimental groups (Fig. S11). Consequently, we selected tail vein injection of 200 μL TCM as the standard condition for establishing pulmonary PMNs.

**Fig. 4:**
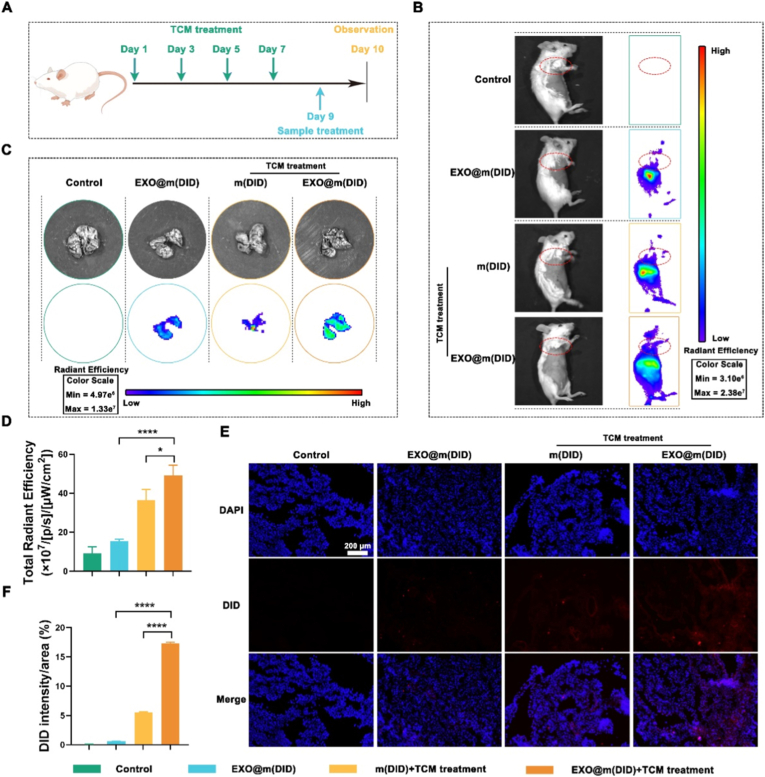
*In vivo* distribution of EXO@m(PDTC) in mice following intravenous injection of TCM. (A) Schematic illustration of the experimental design; (B) Non-invasive NIRF imaging of mice 24 h post-injection; (C) *Ex vivo* NIRF images of lungs harvested from the experimental groups; (D) Quantification of total radiant efficiency in lung tissues; (E) Fluorescence microscopy images of lung sections showing the accumulation of DID-labeled samples; scale bar = 200 μm; (F) Relative DID fluorescence intensity in lung sections as quantified using ImageJ software.

Subsequently, the experimental design is depicted in Fig. 4A. Mice were randomly allocated into four groups. Two groups received 200 μL TCM on days 1, 3, 5, and 7, followed by administration of m(DID) or EXO@m(DID) on day 9. The remaining two groups, serving as normal controls, were injected with PBS or EXO@m(DID) on day 9. On day 10, all mice underwent imaging using the IVIS Spectrum system.

*In vivo* imaging revealed that both m(DID) and EXO@m(DID) displayed targeting affinity for pulmonary PMNs, with EXO@m(DID) exhibiting superior targeting efficiency (Fig. 4B). While m(DID) enrichment relied predominantly on passive targeting, EXO@m(DID) benefited from both passive and exosomal membrane-mediated active targeting. Notably, EXO@m(DID) also generated a detectable fluorescent signal in the lungs of normal mice, further corroborating its active targeting capability. *Ex vivo* lung imaging (Fig. 4C) and quantitative fluorescence analysis (Fig. 4D) aligned with the *in vivo* observations. The total radiant efficiency of EXO@m(DID) was approximately 1.5 times higher than that of m(DID). Fluorescence microscopy of lung tissue sections (Fig. 4E) and subsequent quantification (Fig. 4F) confirmed that EXO@m(DID) achieved the highest enrichment efficiency in lung tissues. It should be noted that coating nanomedicines with exosome membranes does not entirely prevent immune clearance; a substantial portion of EXO@m(DID) still accumulates in organs such as the liver and spleen (Figs. S12 and S13). Therefore, minimizing organ clearance for exosome membrane-coated nanomedicines remains a critical research direction for future studies.

Given the analogous effects of DID and PDTC on nanomedicine surface composition and physicochemical properties, these findings collectively demonstrate that EXO@m(PDTC) possesses robust passive and active targeting abilities toward TCM-induced pulmonary PMNs. In summary, the data indicate that m(PDTC) effectively targets pulmonary PMNs, with enhanced targeting efficiency following exosomal membrane encapsulation.

### 3.5 Evaluation of the anti-metastasis ability of EXO@m(PDTC) in the lung metastasis model established by using intravenous injection of 4T1 cells

**Fig. 5:**
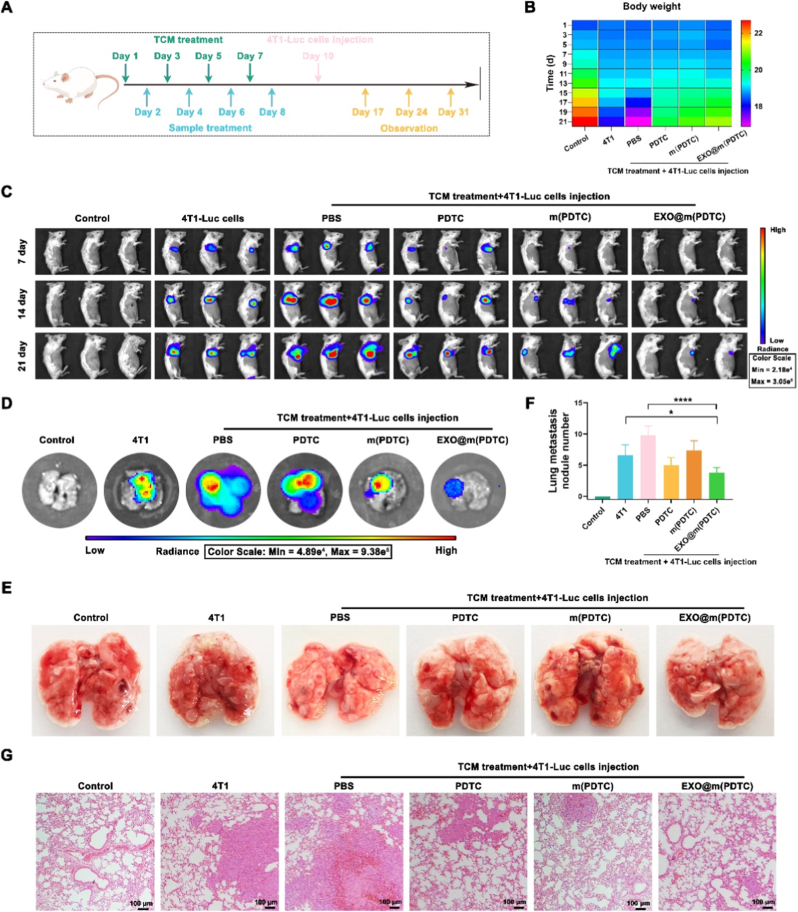
Anti-metastatic efficacy of EXO@m(PDTC) in a mouse lung metastasis model established *via* intravenous injection of 4T1 cells. (A) Schematic overview of the experimental design; (B) Body weight changes of mice in different treatment groups throughout the study period; (C) *In vivo* bioluminescence imaging of lung metastases at 7, 14, and 21 days post-tail vein injection of 4T1-Luc cells; (D) *Ex vivo* bioluminescence imaging of lung tissues collected 21 days after cell injection; (E) Macroscopic appearance of lung tissues from each treatment group; (F) Quantification of metastatic nodules in the lungs; (G) Representative HE-stained sections of lung tissues from each group; scale bar = 100 μm.

To verify the inhibitory effect of EXO@m(PDTC) on pulmonary PMN formation and subsequent breast cancer lung metastasis, we conducted animal experiments as outlined in Fig. 5A. Mice were randomly divided into six groups, including four treatment groups that received both TCM and drug interventions. The treatment protocol consisted of intravenous administration of 200 μL 4T1 TCM *via* tail vein on days 1, 3, 5, and 7, alternating with 200 μL of either PBS, PDTC, m(PDTC), or EXO@m(PDTC) on days 2, 4, 6, and 8 (PDTC dose: 40 mg/kg). Two days after the final injection, all mice received 50,000 4T1-Luc cells intravenously. Control groups received either PBS or 4T1-Luc cells alone on day 10. Pulmonary metastasis was assessed through tumor nodule quantification on days 17, 24, and 31.

Body weight monitoring, performed every two days post-4T1-Luc injection until euthanasia, revealed that while control mice showed significant weight loss following lung metastasis, treated mice exhibited gradual weight gain (Fig. 5B). This observation demonstrated both the biocompatibility of EXO@m(PDTC) and its efficacy in suppressing breast cancer metastasis. *In vivo* bioluminescence imaging confirmed that TCM-induced PMN formation exacerbated lung metastasis compared to direct 4T1 cell injection (Fig. 5C). Although all drug interventions reduced metastasis, EXO@m(PDTC) showed superior efficacy, attributable to its effective PMN inhibition.

*Ex vivo* analyses further validated these findings: bioluminescence imaging (Fig. 5D) showed fewer metastatic lesions in the EXO@m(PDTC) group, while lung tissue anatomy (Fig. 5E) visually demonstrated reduced nodule formation. The total radiance of lung tissues across different groups further confirmed that the EXO@m(PDTC) group exhibited the least lung metastasis (Fig. S14). Furthermore, quantitative analysis revealed an average of 4 metastatic nodules in EXO@m(PDTC)-treated mice versus 10 in controls (Fig. 5F). Histological examination (Fig. 5G) indicated that EXO@m(PDTC) treatment restored near-normal lung architecture, resembling negative controls more closely. Collectively, these results demonstrate that exosome membrane-coated PDTC micellar nanoparticles effectively inhibit PMN formation and breast cancer lung metastasis.

### 3.6 The targeting ability of EXO@m(PDTC) for lung PMN induced by the orthotopic 4T1 tumor

**Fig. 6:**
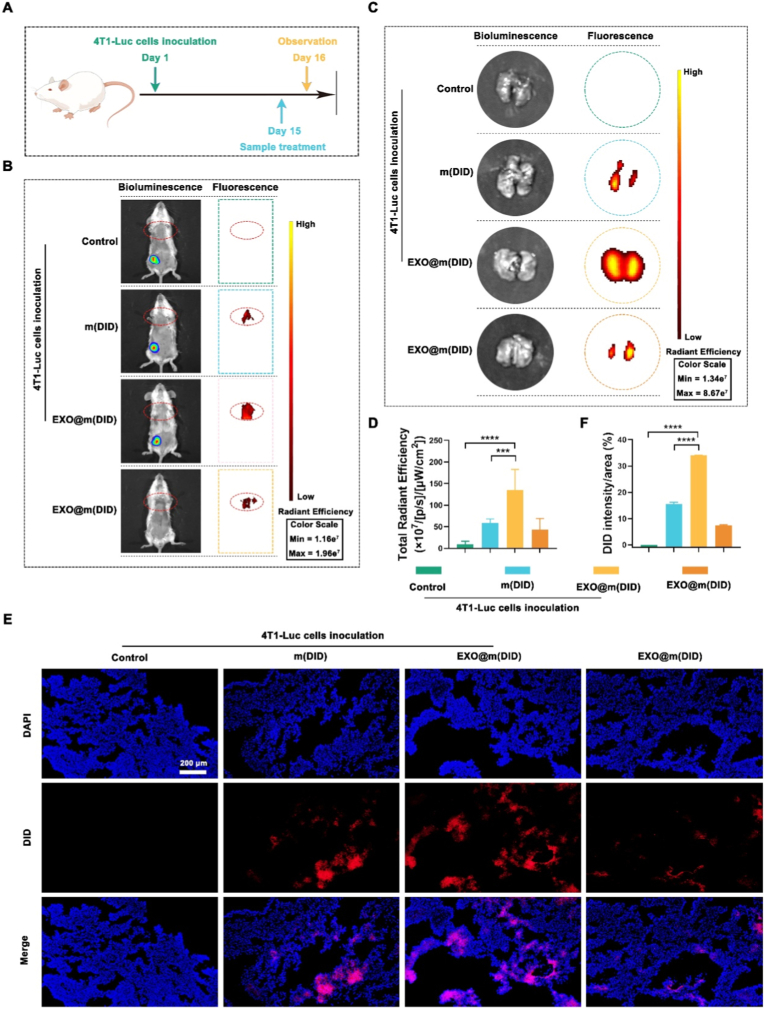
*In vivo* distribution of EXO@m(PDTC) in mice bearing orthotopic 4T1 breast tumors. (A) Schematic illustration of the experimental design; (B) Non-invasive NIRF imaging of mice 48 h after intravenous injection of the sample; (C) Representative *ex vivo* NIRF images of lungs; (D) Quantification of total radiant efficiency in lung tissues; (E) Fluorescence microscopy images of lung sections showing sample accumulation; scale bar = 200 μm; (F) Relative DID fluorescence intensity in lung sections as measured using ImageJ.

In the pulmonary PMN model induced by tail vein injection of TCM, while simulating PMN formation through primary tumor secretions, the influence of the primary tumor itself was neglected when evaluating EXO@m(PDTC)'s PMN targeting capability. To address this limitation, we established an orthotopic breast cancer model to better recapitulate the natural PMN formation process. As illustrated in Fig. 6A, 4T1-Luc cells were implanted into the right fourth mammary fat pad of mice. Fourteen days post-implantation, samples were administered intravenously, with lung accumulation assessed after 48 h. Immunohistochemical analysis, as illustrated in Fig. S15, revealed no detectable levels of CCL-2 or S100A8 in murine lungs one week post-inoculation. Both markers, however, were clearly observed two weeks after inoculation, signifying the initiation of PMN formation. Consequently, targeted assessments were conducted at fourteen days post-implantation to align with this observed biological timeline. *In vivo* imaging (Fig. 6B) revealed primary tumor formation by day 16, though no evident lung metastases were detected. Notably, EXO@m(DID) demonstrated significant lung tissue enrichment, confirming its PMN targeting ability. In addition, m(DID) accumulated in the lungs of tumor-bearing mice, potentially through passive targeting facilitated by PMN-induced vascular permeability enhancement. Consistent with prior findings, EXO@m(DID) also accumulated in healthy mouse lungs. *Ex vivo* lung tissue imaging corroborated these observations (Fig. 6C), with quantification showing EXO@m(DID) accumulation 2.3-fold higher than m(DID) (Fig. 6D). Fluorescence microscopy of lung sections (Fig. 6E) and subsequent quantification (Fig. 6F) further verified EXO@m(DID)'s superior lung enrichment efficiency.

### 3.7 Evaluation of the anti-lung metastasis ability of EXO@m(PDTC) in mice bearing orthotopic 4T1 tumor

**Fig. 7:**
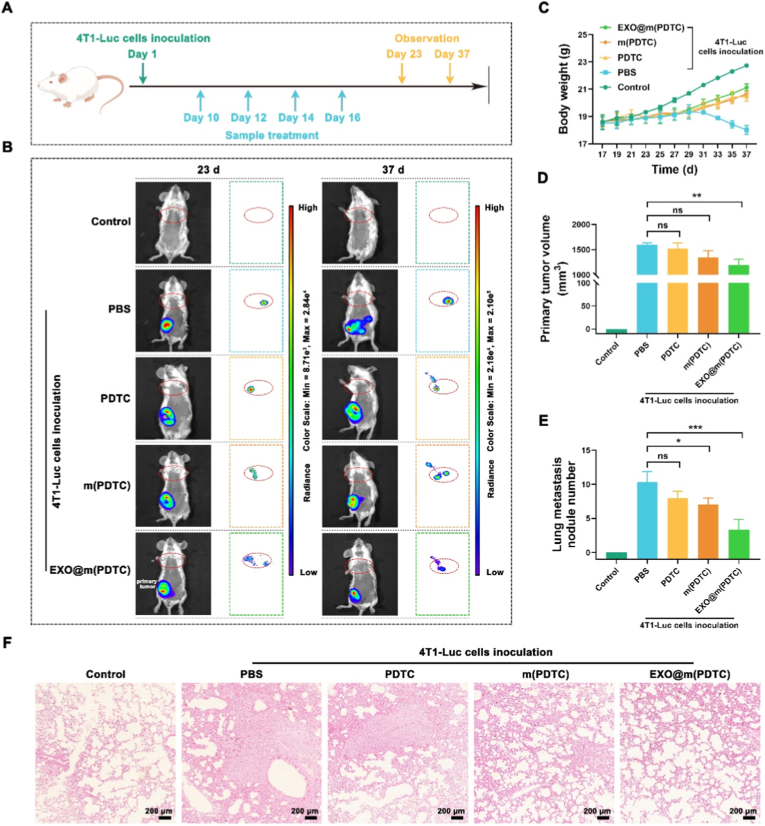
Anti-metastatic effects of EXO@m(PDTC) in an orthotopic 4T1 breast tumor model. (A) Schematic of the experimental design; (B) *In vivo* bioluminescence imaging of lung metastases on days 23 and 37 after orthotopic inoculation of 4T1-Luc cells; (C) Body weight changes of mice in each group during the treatment period; (D) Growth curve of primary tumors throughout the treatment; (E) Quantification of metastatic nodules in the lungs; (F) Representative HE-stained sections of lung tissues from each group; scale bar = 200 μm.

To investigate the inhibitory effect of EXO@m(PDTC) on breast cancer lung metastasis in mice bearing orthotopic 4T1 tumors, we conducted animal experiments as outlined in Fig. 7A. On day 1, 4T1-Luc cells were inoculated, and drug intervention was initiated on day 10 (when tumors reached approximately soybean size) with four treatments administered every other day. Lung metastasis was assessed on days 23 and 37. *In vivo* bioluminescence imaging revealed significant lung metastasis in untreated controls (Fig. 7B), while all drug interventions reduced metastasis, with EXO@m(PDTC) demonstrating the strongest inhibitory effect. Body weight monitoring, performed every two days post-treatment until euthanasia, showed that treated mice gradually gained weight, whereas control mice exhibited marked weight loss following metastasis (Fig. 7C). Primary tumor volumes were also recorded (Fig. 7D), indicating that EXO@m(PDTC) exerted a modest inhibitory effect, likely due to its enrichment at the primary tumor site and suppression of local inflammatory responses [28]. This suggests that precise targeting of PMNs alone may be unrealistic in primary cancer models, necessitating separate regulation of primary tumors and PMNs. Quantitative analysis revealed that EXO@m(PDTC)-treated mice had 2.5-fold fewer metastatic nodules than PBS-treated controls (Fig. 7E). Histological examination (Fig. 7F) showed near-normal lung architecture in the EXO@m(PDTC) group, closely resembling negative controls. Together, these results demonstrate that exosome membrane-coated PDTC micellar nanoparticles effectively suppress lung metastasis in orthotopic 4T1 tumor models, potentially through dual mechanisms involving PMN inhibition and primary tumor interference.

### 3.8 Biosafety of EXO@m(PDTC)

**Fig. 8:**
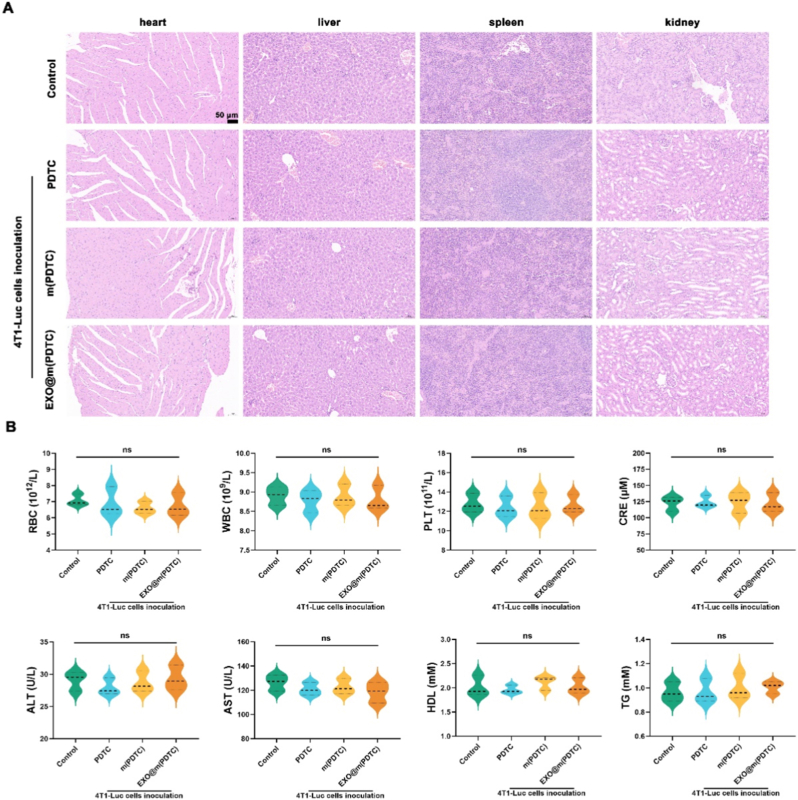
Biosafety evaluation of EXO@m(PDTC) *in vivo*. (A) Representative HE-stained sections of major organs (heart, liver, spleen, and kidneys) from mice in each treatment group; scale bar = 50 μm; (B) Blood biochemical analysis of mice from different treatment groups.

The biosafety of nanomedicines is a critical determinant of their suitability for *in vivo* applications. To evaluate the biosafety of EXO@m(PDTC), mice were euthanized, and their heart, liver, spleen, and kidney tissues were collected for paraffin sectioning. Additionally, mouse serum was collected for the analysis of biochemical parameters. Tissue morphology was subsequently examined *via* HE staining (Fig. 8A). No significant pathological abnormalities were observed in the heart, liver, spleen, or kidney tissues following EXO@m(PDTC) treatment, demonstrating its favorable biosafety profile and potential for *in vivo* use. As shown in Fig. 8B, EXO@m(PDTC) administration had negligible effects on RBC, WBC, and PLT counts in murine serum. ALT and AST levels further confirmed that hepatocellular damage induced by the nanomaterial remained within normal physiological ranges. To assess potential risks of renal impairment and cardiovascular toxicity, we quantified serum CRE as a marker of renal function, as well as HDL and TG, which are sensitive indicators of lipid metabolism. Fig. S16 showed that EXO@m(PDTC) did not induce significant alterations in IL-1β, IL-6, or TNF-α levels in the serum. Collectively, these results demonstrate that EXO@m(PDTC) exhibits no detectable cytotoxicity or adverse effects in mice.

### 3.9 mRNA-Seq data analysis

**Fig. 9:**
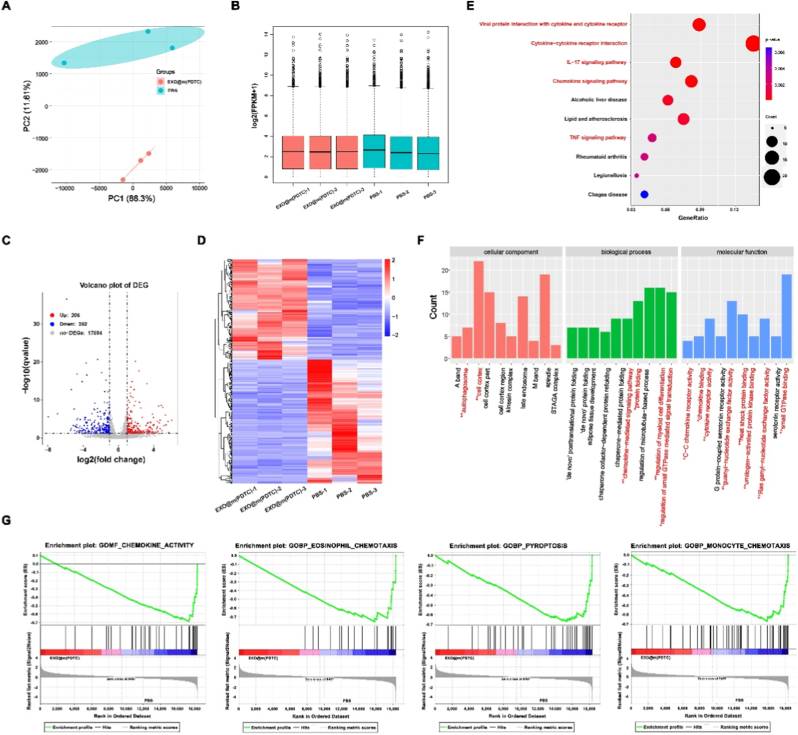
Analysis of mRNA sequencing data. (A) PCA illustrating distinct gene expression profiles among different treatment groups; (B) Box plots comparing the distribution of gene expression levels across groups; (C) Volcano plots of DEGs; Red and blue dots represent up- and down-regulated genes, respectively; FC, fold change; (D) Heatmap displaying expression patterns of selected genes in tumor tissues from each group; (E) KEGG pathway enrichment analysis of DEGs; (F) GO enrichment analysis of DEGs in the biological process category; (G) GSEA of identified gene signatures.

To elucidate the mechanism underlying the inhibitory effect of EXO@m(PDTC) on breast cancer lung metastasis, we conducted transcriptomic analysis of lung tissues from treated mice. Principal component analysis (PCA) (Fig. 9A) and box plots (Fig. 9B) confirmed the reliability of sample clustering and experimental reproducibility. RNA sequencing identified differentially expressed genes (DEGs) with |log2 fold change| > 1.2 and P < 0.05. The volcano plot revealed that EXO@m(PDTC) treatment resulted in 206 upregulated and 252 downregulated genes in lung tissues compared to PBS-treated orthotopic tumor model mice (Fig. 9C). A heatmap (Fig. 9D), accompanied by a dendrogram of gene expression clustering, further highlighted intergroup differences.

Kyoto Encyclopedia of Genes and Genomes(KEGG) enrichment analysis showed that differentially expressed genes (DEGs) were enriched in pathways including “Viral protein interaction with cytokine and cytokine receptor,” “Cytokine-cytokine receptor interaction,” “IL-17,” “Chemokine,” and “TNF” signaling (Fig. 9E). These pathways converge on NF-κB as a central transcriptional hub, forming an interconnected network characterized by a “cytokine to NF-κB to chemokine/cytokine to NF-κB” circuit that regulates multiple tumor-associated physiological functions. In breast cancer lung metastasis, the CXCL12-CXCR4 axis enhances cell invasion *via* NF-κB [29]; TNF signaling sustains inflammatory support in the tumor microenvironment through NF-κB activation [30]; and IL-17 signaling promotes angiogenesis in an NF-κB-dependent manner [31]. Collectively, these interactions drive metastatic progression. Our findings demonstrate that the nanodrug suppresses lung metastasis by targeting multiple upstream regulators of NF-κB, thereby inhibiting the NF-κB signaling cascade.

Gene Ontology (GO) analysis across molecular function, spatial localization, and physiological process further validated the involvement of these pathway-associated molecules in breast cancer lung metastasis (Fig. 9F). Gene set enrichment analysis (GSEA) of transcriptome sequencing results (Fig. 9G) revealed that EXO@m(PDTC) significantly inhibited NF-κB-related pathways, including “Chemokine activity” (NES = −2.45, P < 0.001), “Eosinophil Chemotaxis” (NES = −2.28, P = 0.012), “Pyroptosis” (NES = −2.37, P = 0.002), and “Monocyte Chemotaxis” (NES = −2.57, P < 0.001). NF-κB regulates immune checkpoint molecules such as PD-L1 and modulates the tumor microenvironment's inflammatory state, directly influencing immune checkpoint blockade (ICB) response and serving as a core axis in tumor immunity [32]. IFN-γ amplifies NF-κB signaling by promoting IκBα degradation. The CXCL12-CXCR4 axis, previously shown to recruit tumor cells and induce angiogenesis, was also suppressed by EXO@m(PDTC), as evidenced by the expression heatmap of the GOMF_CHEMOKINE_ACTIVITY gene set [33]. Genes such as Zeb2, Casp3, and Casp8 in the GOBP_PYROPTOSIS gene set further promote metastasis and microenvironment remodeling *via* NF-κB interaction. Transcriptomic analysis confirms that EXO@m(PDTC) downregulates these genes, attenuating their crosstalk with NF-κB and ultimately inhibiting breast cancer lung metastasis.

## 4 Discussion

The development of effective strategies to prevent metastasis remains a central challenge in oncology. Traditional approaches have focused on targeting the primary tumor [34,35]. For instance, by inhibiting the epithelial-mesenchymal transition (EMT) of the primary tumor cells, the migration and invasion ability of the tumor cells can be suppressed [36]. To date, the pivotal role of the PMN in facilitating metastatic outgrowth has shifted therapeutic paradigms toward intercepting metastasis at its earliest stages [37,38]. In this study, we designed a biomimetic nanomedicine, EXO@m(PDTC), which leverages the innate organotropism of tumor-derived exosome membranes to deliver an NF-κB inhibitor specifically to incipient lung PMNs. This strategy diverges from conventional nanoparticle designs and addresses two critical needs in PMN-targeted therapy: precise early-stage accumulation and broad inhibition of inflammatory signaling across stromal cell types.

Our design rationale centers on leveraging the natural biological “address codes” of exosomes. Unlike synthetic nanoparticles or whole-cell membrane coatings [[39], [40], [41], [42]], exosome membranes retain a complex repertoire of adhesion proteins and lipids that mediate organ-specific homing. The successful fabrication of EXO@m(PDTC), confirmed by the retention of membrane proteins and a characteristic anionic surface charge, validates this approach. The near-neutral surface potential of the m(PDTC) core proved crucial for efficient membrane coating *via* extrusion, a practical advantage over some cell-membrane-coated systems, which may require more complex processing to maintain correct orientation. This biomimetic design not only conferred superior targeting to both TCM-induced and orthotopic tumor-induced PMNs but also enhanced cellular internalization across diverse pulmonary stromal cells, a key factor for achieving widespread NF-κB pathway suppression within the niche.

The selection of NF-κB as a therapeutic target is justified by its role as a master regulator of the inflammatory cascade, which orchestrates the formation of the PMN. Our transcriptomic data demonstrate that the anti-metastatic effect of EXO@m(PDTC) is mediated through the coordinated downregulation of a network of NF-κB-dependent pathways, including cytokine-cytokine receptor interactions and chemokine signaling. This pleiotropic inhibition disrupts multiple hallmarks of the PMN, ranging from immune cell recruitment to vascular remodeling. Although the redox-responsive release of PDTC ensures intracellular inhibitor availability, the broader pathway modulation observed suggests that the nanoplatform may exert effects beyond IκBα stabilization, potentially influencing upstream signaling nodes. This underscores an advantage of targeting central regulatory hubs such as NF-κB over individual cytokines. Furthermore, targeting the metabolic reprogramming within the primary tumor or the PMN may represent a novel therapeutic strategy [43].

Despite its demonstrated efficacy, our study identifies critical considerations for translational development. First, while exosome membrane coating significantly enhanced lung accumulation, non-specific uptake by the liver and spleen persisted. This indicates that the inherent tropism of 4T1-derived exosomes, although pronounced, is not absolute. Subsequent iterations could utilize exosomes from lung-tropic cell lines or engineer them to exhibit higher densities of lung-specific targeting motifs to further improve specificity. Second, the observed modest inhibition of primary tumor growth in the orthotopic model suggests that systemically administered EXO@m(PDTC) can also reach the primary site. Although this dual effect is therapeutically advantageous, it complicates the precise delineation of the contributions from PMN inhibition versus primary tumor modulation to the overall anti-metastatic outcome. A potential solution involves developing a dual-nanocarrier system, wherein one vehicle specifically targets the primary tumor and another, optimized for PMN homing, delivers the niche-disrupting agent.

The favorable biosafety profile of EXO@m(PDTC) supports its potential for *in vivo* application. However, the long-term immunogenicity associated with repeated administrations of exosome-based nanomedicines warrants careful investigation, particularly concerning the risk of accelerated blood clearance. Additionally, batch-to-batch variability in exosome membrane composition presents a challenge for scalability. Standardizing isolation protocols and advancing toward engineered exosome-mimetic vesicles may improve reproducibility and facilitate clinical translation.

In conclusion, the EXO@m(PDTC) platform presents a potent and targeted strategy for intercepting metastasis by disrupting the PMN. This underscores the value of biomimetic delivery systems that leverage native biological communication pathways. Future research should prioritize combinatorial approaches, such as integrating PMN disruption with immune checkpoint blockade (ICB) therapy, and employ advanced spatial transcriptomics to map cell-type-specific reprogramming within the niche. These efforts will ultimately facilitate the development of preventive anti-metastatic therapies.

## 5 Conclusion

In summary, we developed a biomimetic nanomedicine, EXO@m(PDTC), designed to intercept breast cancer lung metastasis by targeting its supportive PMN. By encapsulating the NF-κB inhibitor PDTC within micelles coated with tumor-derived exosome membranes, we achieved dual advantages: enhanced active targeting to the pulmonary PMN and improved intracellular delivery. This nanoconstruct effectively suppressed NF-κB-driven inflammatory signaling in key pulmonary stromal cells *in vitro* and demonstrated significant accumulation within PMNs *in vivo*. In both tail-vein and orthotopic metastasis models, EXO@m(PDTC) treatment robustly inhibited the formation of metastatic lung nodules, correlating with downregulation of NF-κB-associated pathways, including cytokine-cytokine receptor interaction and chemokine signaling. Importantly, the therapy exhibited a favorable biosafety profile with minimal systemic toxicity.

This study presents two principal contributions. First, it introduces a novel therapeutic strategy that shifts the focus from treating established metastases to preventing their formation by disrupting the PMN at its inception. Second, it validates the use of tumor-derived exosome membranes as a highly effective targeting moiety for nanocarriers, leveraging natural organotropism to achieve precise drug delivery. Although challenges in scalability and specificity optimization persist, the EXO@m(PDTC) platform provides a promising and versatile foundation for developing next-generation nanotherapeutics designed to intercept metastasis. This work underscores the significant potential of biomimetic approaches in transforming the therapeutic paradigm for metastatic cancer.

## CRediT authorship contribution statement

**Rui Tang:** Investigation, Methodology, Software, Writing – original draft. **Chengyu Mao:** Investigation, Methodology, Writing – original draft. **Caofang Hu:** Investigation, Methodology, Writing – original draft. **Wei Liu:** Investigation, Methodology. **Ju Bai:** Investigation, Methodology. **Yali Wang:** Formal analysis, Resources, Validation, Writing – review & editing. **Lijun Yang:** Conceptualization, Formal analysis, Project administration, Supervision, Validation, Writing – review & editing. **Hongzhao Qi:** Conceptualization, Data curation, Formal analysis, Funding acquisition, Supervision, Writing – review & editing.

## Declaration of competing interest

The authors declare that they have no known competing financial interests or personal relationships that could have appeared to influence the work reported in this paper.

## Data Availability

Data will be made available on request.
