# Supplementary material for: Exosome membrane-biomimetic nanomedicine targets the pre-metastatic niche via NF-κB inhibition to suppress breast cancer lung metastasis
Source: Mater Today Bio. 2026 Jan 31;37:102856. doi: 10.1016/j.mtbio.2026.102856 (PMC12906025; doi:10.1016/j.mtbio.2026.102856)
Supplement: Multimedia component 1 [file mmc1.docx]

**Supporting information**

**Exosome membrane-biomimetic nanomedicine targets the pre-metastatic niche via NF-κB inhibition to suppress breast cancer lung metastasis**

Rui Tang^1,#^, Chengyu Mao^1,#^, Caofang Hu^1^, Wei Liu^4^, Ju Bai^4^, Yali Wang^3,*^, Lijun Yang^2,*^, Hongzhao Qi^1,*^

1. Institute for Translational Medicine, The Affiliated Hospital of Qingdao University, College of Medicine, Qingdao University, Qingdao 266021, China.

2. Qingdao Institute of Bioenergy and Bioprocess Technology, Chinese Academy of Sciences, Qingdao 266101, China.

3. Department of Chemistry, College of Pharmacy, North China University of Science and Technology, Tangshan 063210, China.

4. Department of Emergency Medicine, The Affiliated Hospital of Qingdao University, Qingdao 266003, China.

^#^ These authors contributed equally to this work.


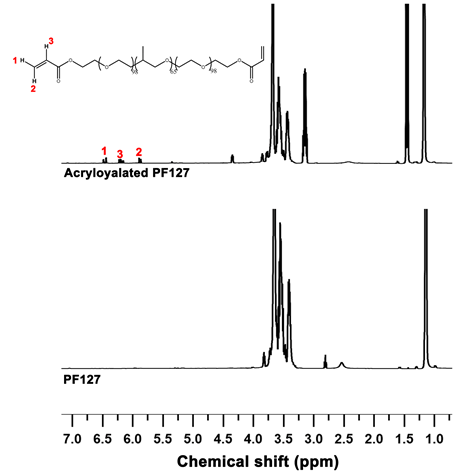


Figure S1. ^1^H NMR spectra of PF127 and acryloylated PF127.


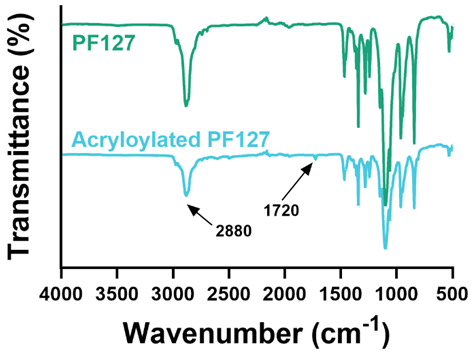


Figure S2. The FTIR spectra of PF127 and acryloylated PF127.


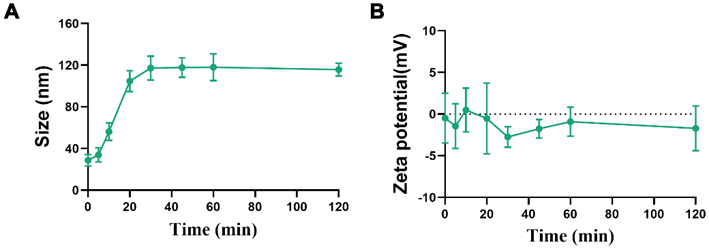


Figure S3. (A) Curve showing the change in m(PDTC) with the extension of polymerization time; (B) Curve showing the change in zeta potential of m(PDTC) with the extension of polymerization time.


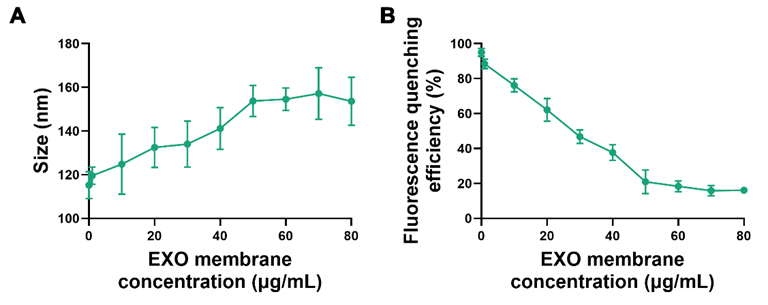


Figure S4. (A) Curve showing the change in the size of EXO@m(PDTC) as the concentration of EXO membrane increases; (B) Curve showing the fluorescence quenching efficiency of FITC-labeled m(PDTC) by anti-FITC antibody as the concentration of EXO membrane increases.


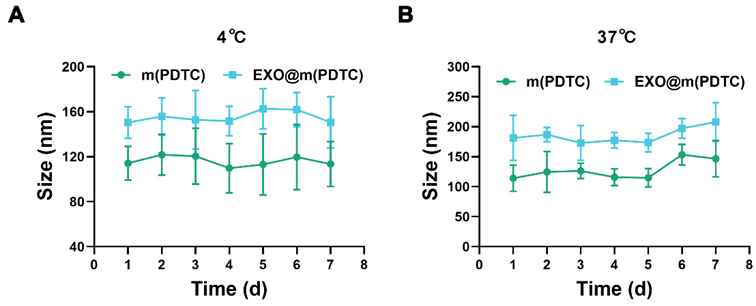


Figure S5. (A-B) Stability assessment of m(PDTC) and EXO@m(PDTC) over 7 days at different temperatures in PBS or DMEM supplemented with 10% FBS, as measured by DLS.


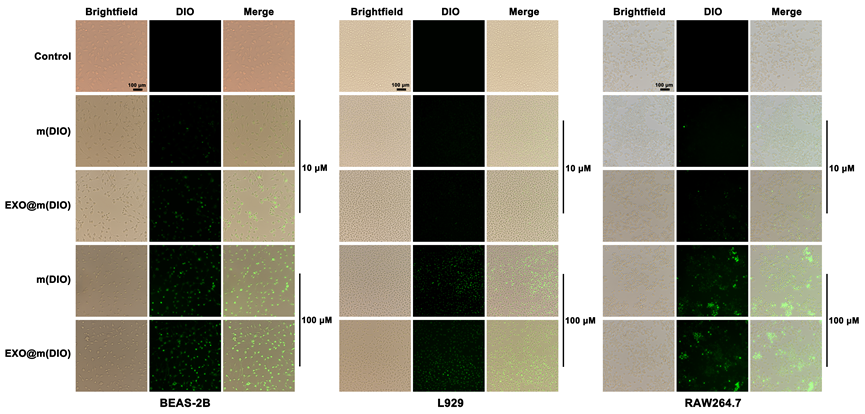


Figure S6. Intracellular distribution of m(DIO) and EXO@m(DIO) in BEAS-2B, L929, and RAW264.7 cells following 8-hour incubation.


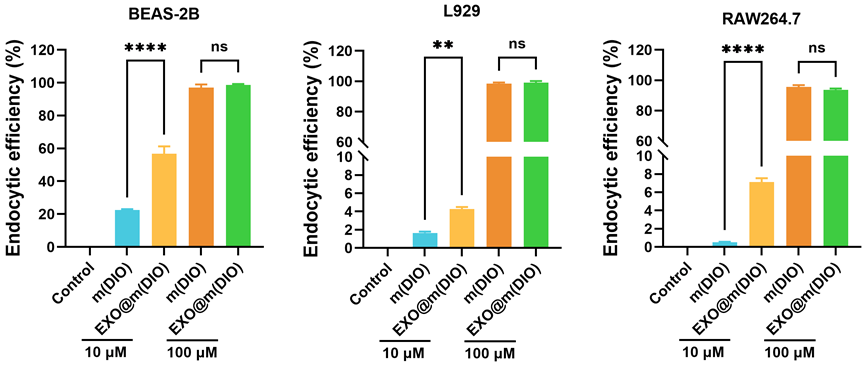


Figure S7. Cellular uptake efficiency of EXO@m(DIO) assessed by quantitative fluorescence analysis.


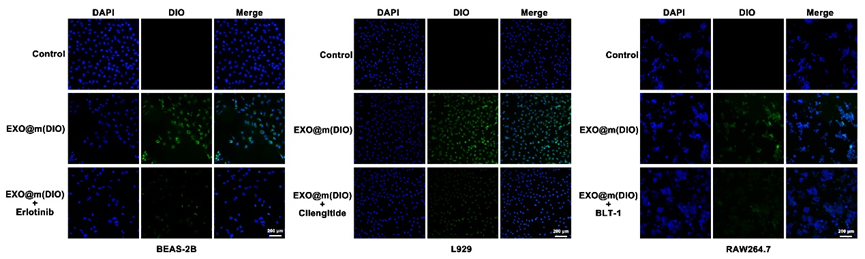


Figure S8. Intracellular distribution of EXO@m(DIO) in BEAS-2B, L929, and RAW264.7 cells following incubation with erlotinib, cilengitide, and BLT-1, respectively.


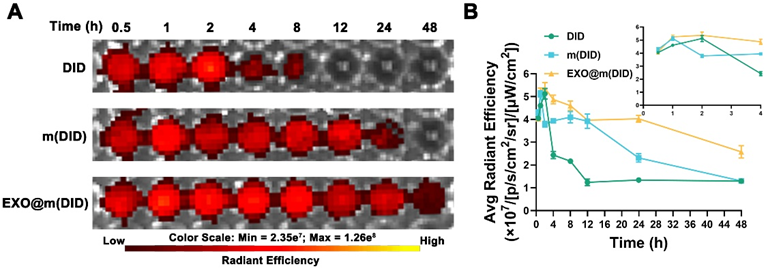


Figure S9. (A-B) *In vivo* pharmacokinetic performance of different formulations after intravenous injection in healthy mice.


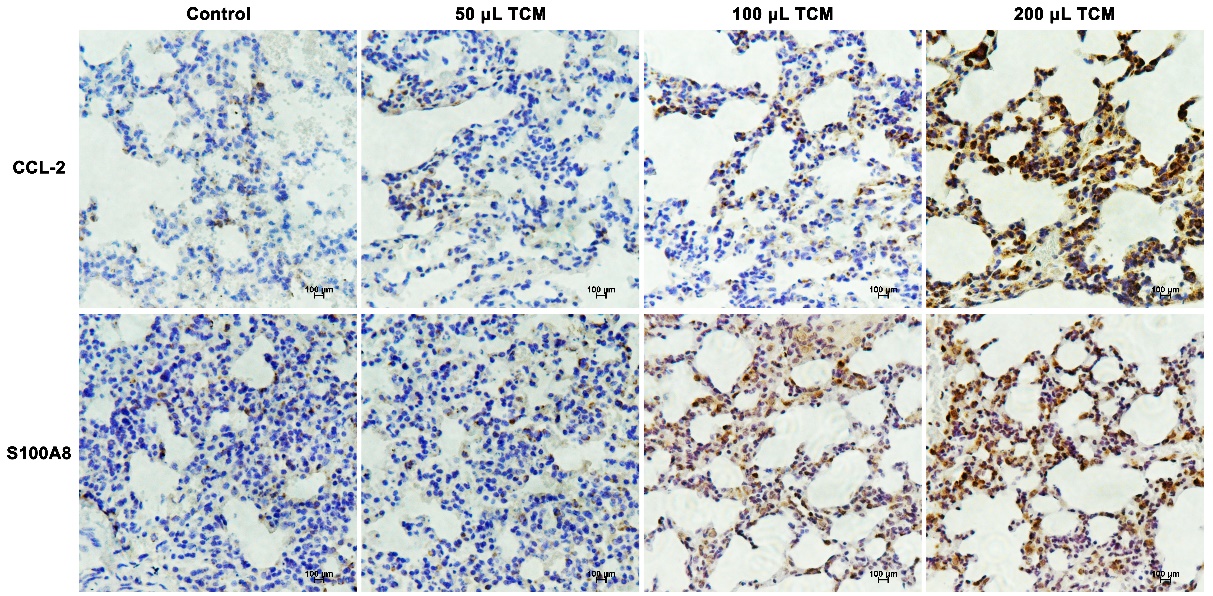


Figure S10. Immunohistochemical analysis of CCL-2 and S100A8 expression in lung tissue from mice administered different volumes of 50% TCM.


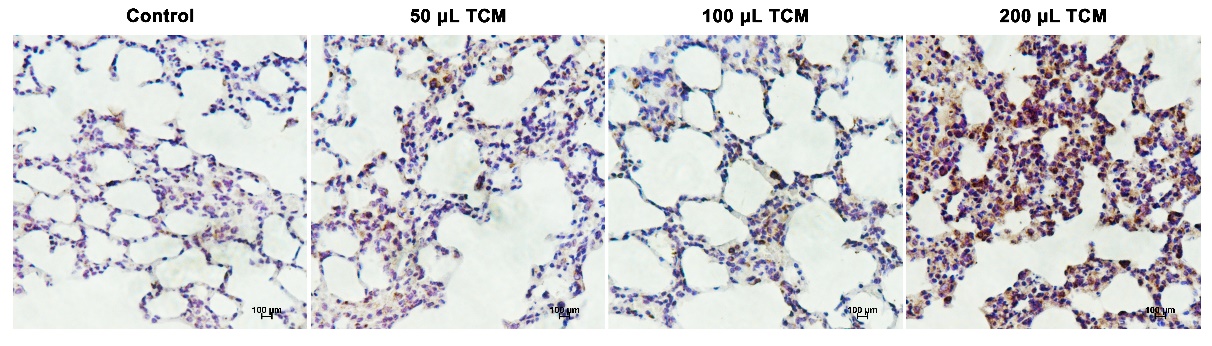


Figure S11. Immunohistochemical staining of IL-1β in lung sections from mice treated with increasing volumes of 50% TCM.


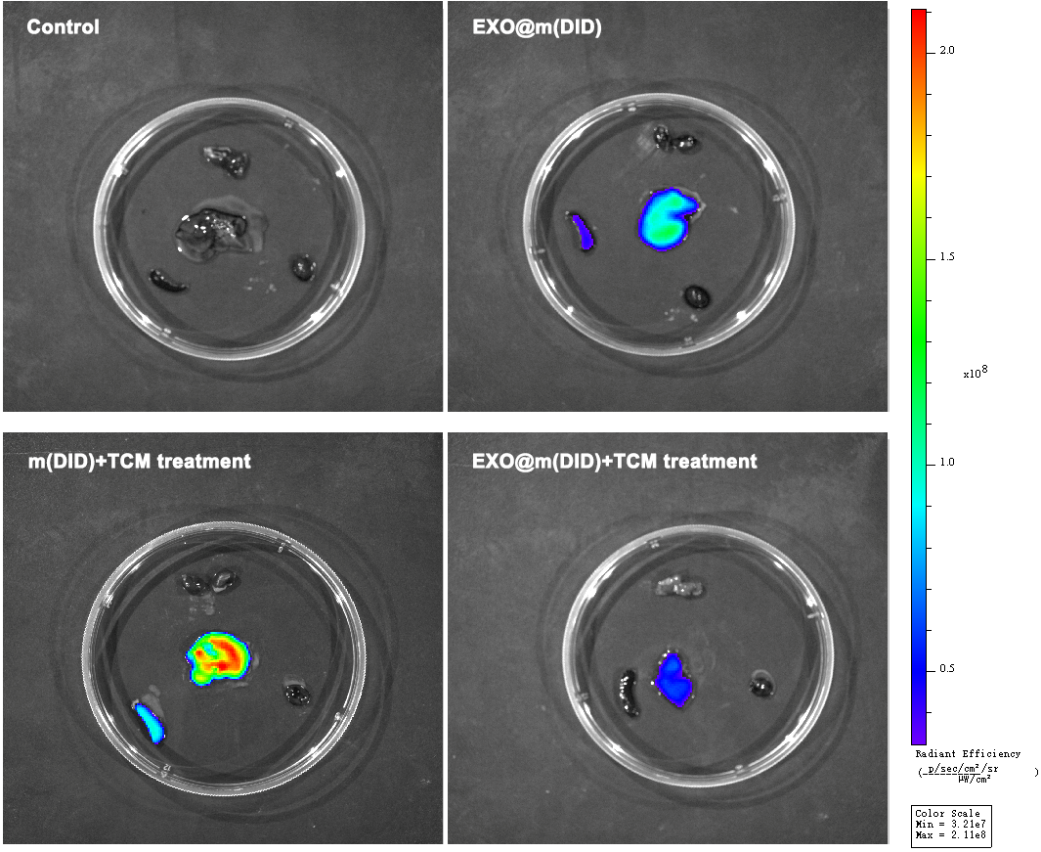


Figure S12. *Ex vivo* NIRF images of the heart, liver, spleen, and kidney harvested from the experimental groups.


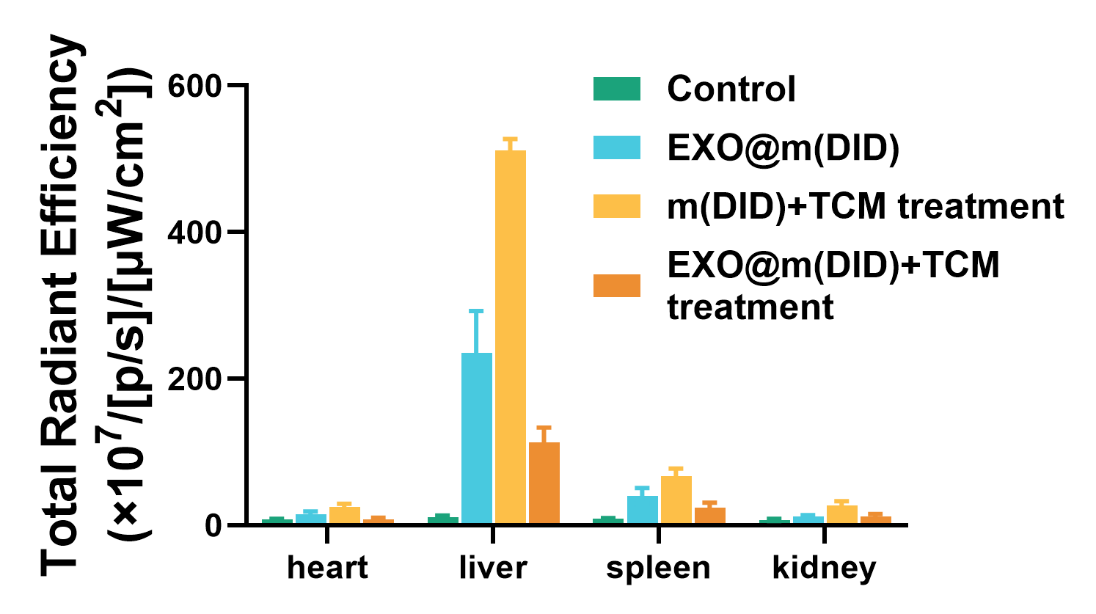


Figure S13. Quantification of total radiant efficiency in the heart, liver, spleen, and kidney tissues.


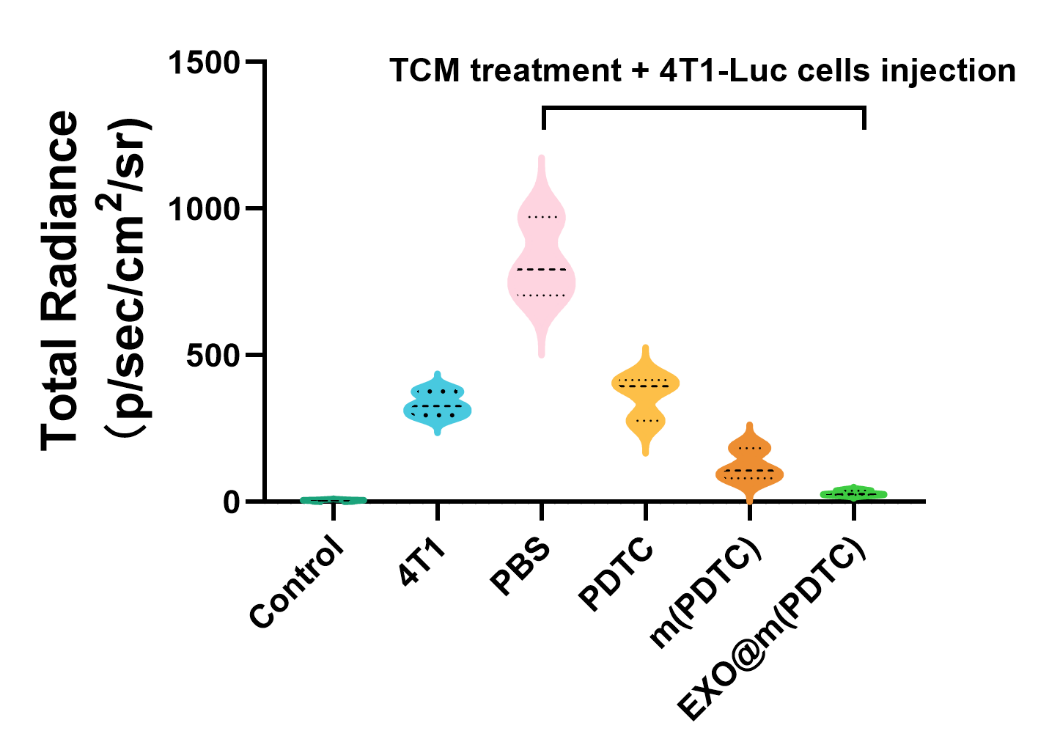


Figure S14. The total radiance of lung tissues across different groups.


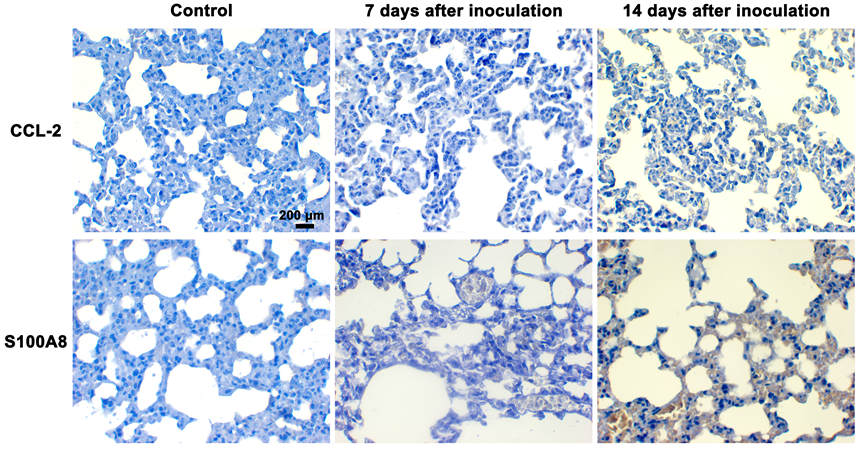


Figure S15. Immunohistochemical analysis of CCL-2 and S100A8 expression in lung tissues from mice bearing orthotopic breast cancer for varying durations.


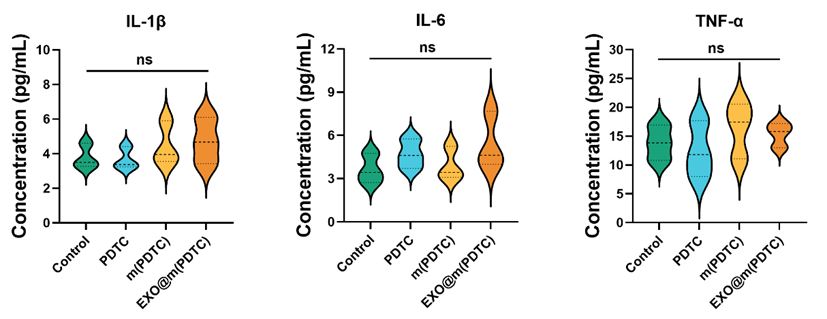


Figure S16. Quantitative determination of IL-1β, IL-6, and TNF-α in serum.

Table S1. Primers and sequences.

| **Primers** | **Sequences** |
| --- | --- |
| IL-1β | F：5’-GCCACCTTTTGACAGTGATGAG-3’  R：5’-ATGTGCTGCTGCGAGATTTG-3’ |
| IL-6 | F：5’-GTCCTTCCTACCCCAATTTCCA-3’  R：5’-TAACGCACTAGGTTTGCCGA-3’ |
| TNF-α | F：5’- CTCCACTTGGTGGTTTGCTAC-3’  R：5’-CTTCCCTCTCATCAGTTCTATGG-3’ |
| GAPDH | F：5’- AGGTCGGTGTGAACGGATTTG-3’  R：5’-TGTAGACCATGTAGTTGAGGTCA-3’ |
